# Power and sample size analysis for longitudinal mixed models of health in populations exposed to environmental contaminants: a tutorial

**DOI:** 10.1186/s12874-022-01819-y

**Published:** 2023-01-12

**Authors:** Kylie K. Harrall, Keith E. Muller, Anne P. Starling, Dana Dabelea, Kelsey E. Barton, John L. Adgate, Deborah H. Glueck

**Affiliations:** 1 Lifecourse Epidemiology of Adiposity and Diabetes (LEAD) Center, University of Colorado - Anschutz Medical Campus, Aurora, CO 80045 USA; 2 Department of Epidemiology, Colorado School of Public Health, Aurora, CO USA; 3 Health Outcomes & Biomedical Informatics, College of Medicine, University of Florida, Gainesville, FL USA; 4 Department of Epidemiology, Gillings School of Global Public Health, University of North Carolina at Chapel Hill, Chapel Hill, NC USA; 5 Department of Pediatrics, University of Colorado School of Medicine, Aurora, CO USA; 6 Department of Environmental and Occupational Health, Colorado School of Public Health, University of Colorado, Aurora, CO USA

**Keywords:** Power analysis, Sample size, Persistent chemicals, Longitudinal study design, Repeated measurements, General linear mixed model, Free software

## Abstract

**Background:**

When evaluating the impact of environmental exposures on human health, study designs often include a series of repeated measurements. The goal is to determine whether populations have different trajectories of the environmental exposure over time. Power analyses for longitudinal mixed models require multiple inputs, including clinically significant differences, standard deviations, and correlations of measurements. Further, methods for power analyses of longitudinal mixed models are complex and often challenging for the non-statistician. We discuss methods for extracting clinically relevant inputs from literature, and explain how to conduct a power analysis that appropriately accounts for longitudinal repeated measures. Finally, we provide careful recommendations for describing complex power analyses in a concise and clear manner.

**Methods:**

For longitudinal studies of health outcomes from environmental exposures, we show how to [1] conduct a power analysis that aligns with the planned mixed model data analysis, [2] gather the inputs required for the power analysis, and [3] conduct repeated measures power analysis with a highly-cited, validated, free, point-and-click, web-based, open source software platform which was developed specifically for scientists.

**Results:**

As an example, we describe the power analysis for a proposed study of repeated measures of per- and polyfluoroalkyl substances (PFAS) in human blood. We show how to align data analysis and power analysis plan to account for within-participant correlation across repeated measures. We illustrate how to perform a literature review to find inputs for the power analysis. We emphasize the need to examine the sensitivity of the power values by considering standard deviations and differences in means that are smaller and larger than the speculated, literature-based values. Finally, we provide an example power calculation and a summary checklist for describing power and sample size analysis.

**Conclusions:**

This paper provides a detailed roadmap for conducting and describing power analyses for longitudinal studies of environmental exposures. It provides a template and checklist for those seeking to write power analyses for grant applications.

**Supplementary Information:**

The online version contains supplementary material available at 10.1186/s12874-022-01819-y.

## Background

Longitudinal epidemiology studies are often conducted in settings where elevated levels of environmental contamination have been detected [1–3]. When an environmental contamination event is discovered and remediated, ideally the exposure is terminated or greatly reduced for the impacted population [4]. However, despite exposure reduction, some contaminants, such as per- and polyfluoroalkyl substances (PFAS), persist for a long time in the bodies of exposed individuals. Health effects may depend not only on the current level of contamination in blood, but also the peak level of contamination experienced, the length of time the internal exposure persists, and the trajectory of the exposure over time. Studies of blood concentrations of persistent environmental contaminants are important first steps toward understanding the health effects of these substances.

To study the amount of time a chemical remains in blood after exposure ceases, scientists often plan a series of measurements from repeated blood samples. Blood levels of chemicals are continuous variables, which can often be transformed to have normal distributions. Such data are often analyzed using general linear mixed models [5], which account for correlation across repeated measurements of blood for each person and can handle missing data. A key part of such a study is conducting a power and sample size analysis.

Accurate selection of the sample size is required to ensure adequate power for detecting associations of clinical relevance or public health significance. The sample size must provide enough power to assess differences in repeated measures of a contaminant, either in people or in the environment. The sample size calculation should also be conducted to match the data analytic approach chosen for the study. This produces an aligned data and power analysis [6]. In addition, the scientist must accommodate other restrictions on design. Exposed populations may be small, and repeated testing of biomarkers can be both difficult and expensive. People living with elevated chemical body burden may be reluctant to participate in repeated sampling, and accurate assessment of biomarker concentrations for many substances of health relevance can cost hundreds of dollars per sample. Environmental health research may also be constrained by the size of the exposed population.

Once a design is proposed, scientists need to assess the statistical power. Power is the probability of rejecting the null hypothesis when the alternative hypothesis is true. Studying power for each study design of interest can help investigators weigh the costs and benefits of enrolling additional participants. Although there are a number of tutorials available that describe the process of selecting sample sizes for longitudinal studies with repeated measures [7–9], the examples are from scientific areas other than environmental health sciences. We give a detailed description of how power and sample size should be calculated, using a planned study of the persistence of PFAS in blood as an example. This work is designed to provide a tutorial on power and sample size, using an example drawn from environmental health sciences.

**Fig. 1:**
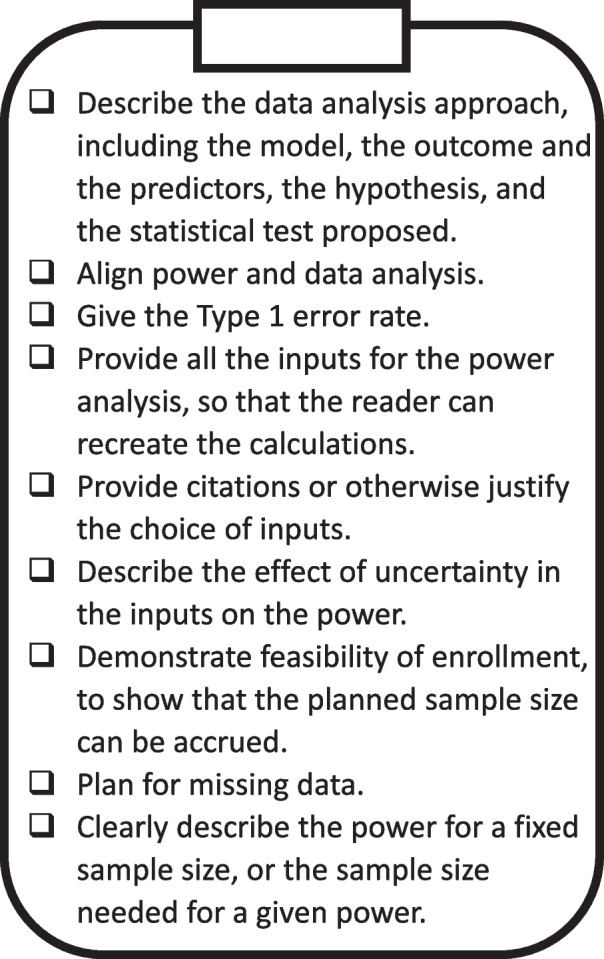
A power analysis check-list

This manuscript is intended for environmental health scientists. We describe the general methodology for a power or sample size analysis for a study with longitudinal repeated measures which will be analyzed with a general linear mixed model. We illustrate how to map study aims to testable statistical hypotheses. We discuss how to match a power and sample size analysis to a data analysis plan, and how to use published inputs to inform power analysis. We recommend methods and software for power and sample size analysis for longitudinal repeated measures. We provide a power analysis check-list for proposal submissions (Fig. 1). We give an example of a power and sample size analysis performed for a grant proposal submission to the National Institutes of Health.

## Methods

### Aligning power and data analysis

Choosing a testable hypothesis and the form of the mixed model is beyond the scope of this paper. Cheng et al. [10] provided a brief and practical set of guidelines for forming a testable hypothesis and selecting model parameters for the longitudinal general linear mixed model. For the purpose of this tutorial, we assume that the statistical analysis plan has specified the hypothesis and the form of the mixed model.

Power analyses cannot be considered until the study design, statistical models and hypotheses are chosen. Without alignment among all three aspects, conclusions drawn from a power analysis are unreliable [6, 11]. Muller et al. 2 (see page 1223, Table 11) gave an example in which computing power for a t-test (column 1, labeled as “Last time”), rather than the more complicated planned analysis with repeated measurements, yielded power which was higher than the true power. If an analyst computes power which is too high, the analyst may mistakenly choose a sample size that is too small. A misaligned power analysis provides the right answer to the wrong question. The sample size may be either too large or too small. Too large a sample size wastes time and money, while too small a sample size increases the chance of missing an important association. While exact alignment may be unattainable, a key goal is to obtain the closest alignment possible. Accurately accounting for correlation across repeated measurements is an important consideration. Good alignment requires the power and sample size analysis to use the same statistical test, model, and assumptions about the correlation and variance patterns as the planned analysis of the data. Equally importantly, the assumptions of the planned analysis must properly align with the actual data.

### Methodology for computing power for longitudinal models

Approaches to computing power for the longitudinal general linear mixed model fall into four clusters [12]. Power and sample size methods can be characterized as 1) approximations only guaranteed to be accurate in large samples [13–18], 2) exemplary data approaches [19, 20], 3) simulation methods [21–24], or 4) approximate (or exact) methods that are accurate in both small and large sample sizes.

We recommend using approximate or exact methods that are accurate in both small and large samples sizes [6]. Our rationale is based on arguments from Chi et al. [12]. Chi et al. [12] noted that large sample approximations may give misleading high or low power values for the relatively small sample sizes of many studies of environmental contamination. The problem with large sample approximations for power is that the accuracy depends on the sample size, the hypothesis, and the study design in a complex way. It is better to use a power approximation method which is accurate across a broad swath of designs [6]. Chi et al. [12] demonstrated that the exemplary data approach may not apply for missing and mistimed data, which is common among environmental epidemiology studies with repeated measurements and longitudinal designs. Finally, Chi et al. [12] pointed out that simulation-based approaches for power analysis should meet software-industry standards, including testing and documentation.

### Power analysis software

Computing power or sample size for the longitudinal general linear mixed model requires software to handle the multiple inputs and complex approximations. Given the rapid changes in software, it is impractical to list all software packages that offer power and sample size computations for studies of correlated longitudinal repeated measures.

There are three software packages that implement the methods preferred by Chi et al. [12] Two commercial software products, PASS [25], and SAS PROC POWER [26], implement the preferred methods, but require license fees. We recommend GLIMMPSE [27, 28], which is free, open-source, point-and-click, and requires only a web-browser to use.

The description of GLIMMPSE [27] in the Journal of Statistical Software includes extensive validation studies, which compare the accuracy of the software to Monte Carlo simulations. The web site hosting GLIMMPSE also links to related power and sample size publications, tutorials, a manual, and citation information. In addition, the site links to material from a short course funded by R01GM121081 and taught at five universities to over 240 learners. GLIMMPSE was developed with funding from five National Institute of Health (NIH) grants. GLIMMPSE allows the user to input either LEAR or unstructured covariance structures to account for correlation between repeated measurements, supports interaction hypothesis tests, and supports a wide variety of statistical tests such as the Wald test. GLIMMPSE also allows the user to assess sensitivity by calculating power over different scales of the mean difference and standard deviation. The GLIMMPSE software will also compute confidence intervals for power calculations based on estimated variances and correlations.

### Obtaining inputs for power analysis

Validated software products will produce reasonable power and sample size numbers, given the appropriate inputs. But finding such inputs is often the most difficult part of power and sample size analysis. For a power analysis of a longitudinal study with continuous repeated measures, one needs multiple inputs. Required inputs include 1) the predictors in the model, 2) the Type I error rate, 3) a clinically significant difference or minimally detectable difference in the outcome, 4) the variances and correlations of the outcome variables, given the predictors, and 5) the choice of the hypothesis test. If sample size is fixed, investigators are seeking to predict power or confidence interval width. Otherwise, investigators must specify the power desired for a study, and choose a sample size large enough to attain that power.

Environmental exposures often affect small populations, fixing the sample size. The goal shifts to computing power for a mean difference of interest. The choice of a mean difference of interest depends on scientific concerns intrinsic and extrinsic to the experiment. One approach for calculating power uses a hypothesized difference that is driven by clinical, environmental, regulatory, or public health concerns. In some cases, a current regulatory standard may determine a mean difference of interest. In other cases, it may be known what differences in levels of environmental contaminants are associated with clinically important health effects. When no previous studies have determined a clinically relevant difference, another approach is to find the smallest difference that can be observed at a predetermined power.

Scientists turn to three main sources for finding the remaining power inputs. They include separate pilot studies, internal pilot designs, and published sources. The goal is to find reasonable choices for mean values for each predictor group at each time point, and corresponding values for correlations and standard deviations of outcomes, adjusted for the predictors.

A pilot study is a small study conducted before the main study. Results from the pilot study may be used to provide inputs for power and sample size analysis of the main study. However, separate pilot studies often require separate funding. In addition, for an environmental contamination event, the time required to run a pilot study may allow internal contaminant concentrations to drop, preventing timely ascertainment of peak exposure.

As an alternative to traditional pilot studies, an “internal pilot design” may be used. An internal pilot design uses data collected from the first few participants to estimate the standard deviations and correlation in order to choose a sample size. As an adaptive design, the approach requires careful planning to control Type I error rate [29].

If a pilot study is not practical, a systematic literature search is the best option to find inputs based on data. The search begins by finding studies as closely comparable to the planned study as possible. There is often a choice of published studies on which to base inputs. Studies can be considered comparable if populations, outcomes, study time frames and covariates are similar. For a longitudinal repeated measures design, comparability requires similarity in the timing of the repeated measures. Analysts should cite the published studies from which they extracted inputs. If there are many published papers, and the inputs from the papers differ, the analyst should describe their choice and provide the rationale.

Unless the planned study is a replication of a previous study, it is unlikely that any study in the literature will be an exact match to the planned study. It is important to recognize that ethical power analysis is a good-faith attempt to do the best one can, an attempt that should be reported in detail, including the limitations. Power analysis is often, at best, an informed guess as to what a future study will find.

Selecting inputs for power analysis is not exact. When using statistical estimates as inputs, there are approaches to help account for statistical uncertainty [30–32]. The methods give confidence intervals around the power values and sample size for the study proposal. Another approach is to conduct a sensitivity analysis. A sensitivity analysis includes alternate values for mean differences, variances and correlations. An analyst may consider values for means and variances twice as big, or half as big, as the original hypothesized value, and examine the effect on power. A table of alternative inputs, and the associated sample sizes, is useful.

The most difficult values to obtain from published reports are estimates of correlations among repeated measures, and changes in variance across time. Failing to account for within-participant correlations can lead to incorrect power and sample size calculations (Muller, LaVange, Ramey and Ramey 1992). A common error occurs when analysts assume a simple pattern in correlation, when the true pattern is complex [33]. Fitting an unstructured covariance matrix avoids the error of oversimplification.

In innovative studies which are first in their field, there may not be information about covariance structures available in the literature. In these cases, one possibility is to consider using a Linear Exponential Auto Regressive [34], which uses a small number of parameters to specify a correlation pattern that decreases over time. If the correlation pattern was chosen speculatively, the analyst should make this clear when reporting the power analysis. If the Linear Exponential Auto Regressive is biologically implausible, there are many other correlation structures available which an analyst could specify for a power analysis. Littell et al. [35] provide a nice summary of options. Some authors suggest that a conservative approach is to try different covariance structures, compute the sample size required for adequate power for each covariance structure, and choose the largest sample size required [36, 37].

For longitudinal studies, an accurate power analysis should allow for missing data and attrition. Failing to do so when needed can lead to an unrealistically small choice of sample size [38]. The issue is magnified in longitudinal studies in which participants are required to attend multiple visits. Previous studies in similar populations can provide realistic estimates for dropout, missing data and attrition [39].

### Recommendations for writing a power analysis

In Fig. 1, we summarize recommendations for writing power analyses [6–8, 27]. As recommended by Gawande [40], we provide instructions in the form of a checklist. Including all the elements in the checklist allows the reader to recreate the power analysis themselves. The ability to check power calculations extends the idea of reproducibility in science [41] to study design.

**Fig. 2:**
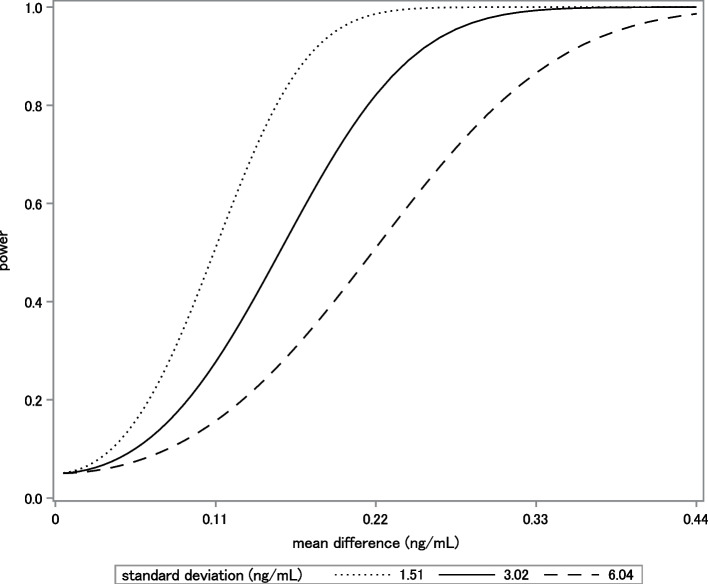
Power for time-by-group interaction, as a function of mean difference [(μ T1,A - μ T3,A) -(μ T1,C- μ T3,C)] and standard deviation of PFHxS. Data are back-transformed for interpretability. The standard deviation shown is 3.02 ng/mL. This mean difference is scaled by factors of 0.5, 1, 1.5, and 2 along the x-axis. The solid line shows how differences in the mean difference may affect power. Additionally, the two dashed lines show how the relationship between the mean difference and power changes when the standard deviation is smaller by half (1.51 ng/mL) or doubled in size (6.04 ng/mL)

A power curve, such as that shown in Fig. 2, is often helpful. The curve shows the sensitivity of the power analysis to changes in the input values. Both changes in mean differences and standard deviations affect power.

## Results

### Overview

We use a power analysis for a longitudinal study of blood PFAS concentrations as an example. We describe the background, the statistical analysis plan, the hypothesis tests, a power analysis that matches the statistical analysis plan, the inputs for the power analysis, and provide an example power analysis. We also provide step-by-step screenshots for the power analysis (Additional file 2).

### Background for an example longitudinal study

PFAS are a class of chemicals that have been widely used for more than 70 years in industrial and commercial applications due to their unique surfactant properties [42]. PFAS are used in numerous consumer and industrial applications, including stain- and water-resistant textiles, non-stick cookware, and fire-fighting foams, as well as specialized applications in electronics, photography, and hydraulic fluids [43, 44]. Humans are exposed to PFAS through a variety of routes, but in the general population, exposure is most frequently via ingestion of contaminated food and water [45–49].

No national enforceable standards have been set for PFAS in drinking water. However, the United States Environmental Protection Agency (USEPA) has established health advisories (70 ng/L) for two commonly measured PFAS, perfluorooctane sulfonate (PFOS) and perfluorooctanoate (PFOA) [50, 51]. The health advisories were developed based on results from animal and epidemiologic studies which show an association between PFAS chemicals and developmental toxicity, carcinogenicity, as well as potential adverse effects on liver, immune, and endocrine function [52].

Between 2013 and 2015, PFAS concentrations above the USEPA health advisory were detected in public water systems in the towns of Fountain, Security and Widefield, all located in El Paso County, Colorado. Combined, the three water systems as well as local private wells serve approximately 70,000 people. These water sources were likely contaminated years before 2013, although when the contamination first reached local water supplies remains unclear. The contamination likely occurred as the result of the use of aqueous film-forming foams (AFFF) for firefighting at Peterson Air Force Base. Studies have shown that the concentration of PFAS decreases as the groundwater flows away from the air force base [53–55]. A preliminary survey (R21 ES029394, PI: Adgate) of blood concentrations of PFAS in 213 local adult residents showed that participants had median blood concentrations of perfluorohexane sulfonate (PFHxS) and PFOS roughly 12 and two times, respectively, as high as the median of the U.S. general population (Barton et al. 2019). While PFHxS is structurally similar to PFOS, studies indicate it has a longer elimination half-life in humans [56, 57].

Following the discovery of PFAS concentrations above the USEPA health advisory, the Water Authorities of Fountain, Security and Widefield moved to change sources and implement water treatment to reduce the concentrations of PFAS reaching consumers. According to the Colorado Department of Public Health and Environment, the best estimate of when consumers in Fountain were last exposed to high PFAS concentrations in drinking water was August of 2015. Security and Widefield were exposed to high concentrations, at least sporadically, until summer 2016.

#### Goals of the planned study

The complex mixture of PFAS present in the contaminated water has not been fully characterized. The water contains both frequently measured and previously uncharacterized PFAS. The rate of excretion of some of the various components in this mixture is unknown. The goal of the proposed study will be to describe the rate of decline in blood concentrations over a three-to-five-year period in adults and children.

#### Study design

We describe the study design for which we computed power. We proposed a longitudinal repeated measures study design. Study participants would be recruited from those exposed to PFAS-contaminated drinking water in Fountain, Security and Widefield, and would give written informed consent. During the three-year study, we would collect three, approximately equally spaced, repeated blood samples from 500 adults and 500 children for PFAS quantification. These would occur at approximately four, five, and 6 years after contamination ended. To avoid clustering within family units, we would only allow one study participant to enroll per family unit.

We chose the sample size based on two factors. First, the study investigators had conducted a pilot study in the population of interest, and so they had an estimate of PFAS blood concentration, the size of the eligible population, and the recruitment rate they could expect. Second, the maximum budget limited the number of samples. Because the sample size was fixed by cost constraints, the investigators sought to compute power.

#### Methods for the planned study

In the planned study, all study participants would have blood collected three times during the study period. A panel of PFAS (PFHxS, PFOS, PFOA) would be measured in blood using a High Performance Liquid Chromatography Turbo Ion Spray ionization tandem mass spectroscopy instrument with isotopic dilution [58]. The limits of detection for all PFAS are approximately 0.1 ng/mL. The precision and accuracy of the estimation ranges from 5.1–15.4% and 87–108%, respectively [58].

### Translating goals to hypotheses

Study investigators wished to compare blood concentrations of PFAS between children and adults. They hypothesized that the rate of decline in blood PFAS concentration over time would differ between adults and children. This hypothesis corresponds to examining the strength of the time-by-life stage interaction.

### Choosing a modeling approach

The investigators planned to use a linear model, a similar approach to that used in Olsen et al. [56]. The rationale for using a linear, rather than non-linear model follows. Thompson et al. [59] gave a single compartment pharmacokinetic equation for modeling blood concentrations of PFOA and PFOS. We assume that PFHxS will follow a similar model. The water supplies of Fountain, Security, and Widefield have remediated to concentrations below the USEPA health advisory, so there is assumed to be little continued exposure via drinking water. Modifying the equation of Thompson et al. [59] implies that the logarithm of the concentration in blood is linear in time, an observation corroborated by the findings of Olsen et al. [56] and others. Since the log of blood concentration followed a linear model, the investigators chose to use three repeated measures of the log of concentration as the outcomes in the model.

To account for potentially missing and mistimed data, and allow for repeated measures in a longitudinal study, the investigators planned to use a general linear mixed model [5], as opposed to a multivariate model, which would not accommodate missing data. A linear exponential autoregressive covariance structure would be included to account for repeated measurements within participants. The investigators chose a linear exponential autoregressive covariance structure, because they felt that the correlation between measurements would decrease slowly across time. The Wald test with Kenward-Roger degrees of freedom would allow for hypothesis testing with an accurate Type I error rate [60, 61].

As predictors in the model, the investigators chose to use an indicator variable for stage of life (child or adult). They defined adulthood as the onset of puberty (menarche for girls and pubic hair for boys) [62]. Why use an indicator variable, instead of using age as a continuous predictor? As children grow during and after a contamination event, their body size and blood volume increase, diluting the concentrations of the chemical. Growth in body volume and hence blood volume in time is roughly linear across the age group of children to be studied [63]. The linear growth means that the dilution factor per year of age is the same, no matter the age of the child at the contamination event. Thus, children can be considered a homogenous group in terms of their change in blood concentrations over time after the contamination event.

### Aligning power and data analysis

The planned data analysis used a linear mixed model that accounted for correlation between repeated measures of the outcome. The planned hypothesis testing approach was to use a Wald test to examine the time-by-life-stage interaction. An aligned power analysis needs to assume a similar model, hypothesis, and hypothesis testing approach. The investigators chose to perform this power analysis using GLIMMPSE [27], which computes power for the longitudinal studies analyzed with general linear mixed models.

### Obtaining inputs required for power analysis

A small cross-sectional pilot study was conducted, and provided blood measurements of PFOS, PFHxS, and PFOA collected in June 2018 [4]. This date was approximately two-years after the contamination event ceased in all three towns. These data provided an estimate of blood PFAS concentrations in the exposed population. Because the pilot study was cross-sectional, it did not provide estimates of correlation between the repeated measures, nor did it describe the pattern of decline of PFAS in blood over time. Choices for decline over time and correlations among repeated measures are needed to conduct a power analysis for the proposed longitudinal design.

To select the correlation values and the rate of decline over time, we conducted a systematic literature review. A search for “perfluorinated compounds” or “perfluoroalkyl substances” in PubMed resulted in more than a thousand publications. By filtering the collection to find highly cited longitudinal studies, we condensed the publications down to five options [1, 56, 57, 64, 65]. We then searched the remaining five publications for repeated measurements, a similar length of follow-up as the proposed study, a similar time since the exposure ended, and a similar profile of PFAS exposure in each publication.

In this case, we chose a study from Olsen et al. [56] on which to base the inputs for the power calculation. The paper was chosen for two reasons. First, the paper has been cited 752 times (Web of Science, 9/25/2020). Second, the USEPA used the paper to develop the current health advisories [66, 67]. Olsen et al. [56] followed their cohort for an average of 5 years, a similar length of follow-up to the proposed study. In addition, the cohort in the Olsen et al. [56] paper had a similar profile of PFAS exposure to the proposed study, in that the highest blood concentrations were detected for PFOA, PFOS, and PFHxS. The contamination in the Olsen et al. [56] paper ended within a similar time frame as the proposed study, about 0.4–11.5 years before the study began. Finally, Olsen et al. [56] collected up to eight blood samples for each participant, and published enough detail to allow readers to infer the correlation structure of the log PFAS blood concentrations.

There were some differences between the study described in Olsen et al. [56] and the proposed study design. Olsen et al. [56] had an occupational cohort, so nearly all study subjects had initial PFAS blood concentrations higher than expected in the proposed study population. It is possible that the differences in populations may lead to a difference in the rate of elimination of PFAS from the blood. Further, the study from Olsen et al. [56] only reported data for an adult cohort of mostly males. There are clear biological differences between adults and children, such as growth, which meant that the investigators of the proposed study had to decide how to extrapolate power inputs for children.

#### Selecting inputs: decline in PFAS over time

In Fig. 1 (pages 1302–1303), Olsen et al. [56] showed that there was a log-linear relationship between time and PFAS concentrations in blood, during the 5 years the participants were studied. Although Olson does not report the slope of the lines, they do provide enough data to calculate them. The rate of change in PFAS concentration over time can be computed using log transformations of the reported PFAS concentrations, and the number of days between the initial and final measurements, excerpted from Olsen et al.’s [56] Table 2.

Values for adults at approximately two-years post contamination in the Fountain, Security and Widefield population were obtained from Barton et al. (2019). Projected values for the Fountain, Security and Widefield population at four, five, and 6 years post-contamination were projected from log transformations of the observed starting values, assuming that the decline in concentration was the same as that observed, on average, in Olsen et al. (2007). The interpolated values for PFHxS are shown in Table 1.

**Table 1:** Speculated means and standard deviations of PFHxS blood concentrations (ng/mL), and the associated log transformed inputs for power analysis

	Untransformed (ng/mL)				Log-transformed (log ng/mL)			
Life-stage	Standard deviation	4 years	5 years	6 years	Standard deviation	4 years	5 years	6 years
Children	3.02	4.57	3.80	3.24	0.48	0.66	0.56	0.51
Adults	3.02	11.22	10.23	9.12	0.48	1.05	1.01	0.96

#### Estimating inputs: standard deviation of PFAS from pilot

Multivariate linear models were used to estimate the standard deviation of each PFAS. The outcome was estimated log transformed PFAS blood concentration at years 4, 5, and 6. The predictor was the model intercept. The standard deviations were estimated using residuals from the model.

#### Extrapolation of response variables for children

With the assumption that adults and children excrete PFAS at the same rate each year, we developed the following plan to predict PFAS concentrations for children on a year-to-year basis. Children consistently grow in weight (and thus volume), so we needed to account for the dilution effect that growth would have on each estimate of blood PFAS concentration. Between 3 and 11 years of age, children gain weight in an approximately linear fashion [68, 69]. Children have an average 15% increase in weight per year, meaning that we needed to adjust for a 60%, 75% and 90% increase in weight at four, five and 6 years post-contamination, respectively. Assuming that 1 kg of growth in weight is roughly equal to 1 L growth in volume, we estimated PFAS concentrations for children while accounting for growth in volume. The computations used an equation for dilution (concentration_1_*volume_1_ = concentration_2_*volume_2_). Results may be seen in Table 1.

#### Estimating inputs: correlations

Longitudinal correlations were calculated for PFAS using initial, day 730, and final measurements from Olsen et al. [56]. The observed correlation coefficients best fit a LEAR model [34], with a base correlation of 0.9 and a decay rate of 1.0. Coefficients for the LEAR model are shown in Table 2. An advantage of fitting a correlation pattern model is that it provides estimates of correlations for all measurements for the proposed study.

**Table 2:** Speculated within participant LEAR correlation matrix (base correlation = 0.9, decay rate = 1)

	Time 1	Time 2	Time3
Time 1	1.00	0.90	0.81
Time 2	0.90	1.00	0.90
Time 3	0.81	0.90	1.00

#### Demonstrating the effect of uncertainty on power

Power curves summarize the dependence of power on inputs. The example discussed in the paper is a test of interaction. Interaction can be conceptualized as a test of two differences of differences: A) [(μ_T1,A_ – μ _T2,A_) – (μ_T1,C_ – μ_T2,C_)], and B) [(μ _T1,A_ – μ _T3,A_) – (μ _T1,C_ – μ _T3,C_)]. Here, μ_T1,A_ – μ_T2,A_ represents the difference in mean response for adults between time 1 and time 2. Similarly, μ_T1,C_ – μ_T2,C_ represents the difference in mean response for children between time 1 and time 2. T3 is used to indicate the third time point. It is convenient to parameterize the test so that the first term, [(μ_T1,A_ – μ_T2,A_) – (μ_T1,C_ – μ_T2,C_)], is zero, and the second term is non-zero. This can be done because the adult versus child comparison only involves two groups. The reparameterization allows plotting the power curve as a function of the second non-zero term only. Fig. 2 shows [(μ_T1,A_ – μ_T3,A_) – (μ_T1,C_ – μ_T3,C_)] on the x-axis, and the power on the y-axis. Figure 2 also shows how changes in standard deviation may impact power. The three lines shown are the power if we observe the standard deviation we expect (in the middle), and if we see half or twice that standard deviation (on top, and bottom, respectively).

### An example power analysis

A power or sample size analysis should contain all the information needed for a reviewer to recreate the results. We give an example of power analysis in the next paragraph. The power analysis follows the checklist given in Fig. 1.

The power computations assumed a longitudinal study analyzed with the general linear mixed model. Outcome variables were three repeated measurements of log PFHxS concentrations over time. The predictor was a categorical variable that distinguished adults from children. Investigators planned to test for time-by-life-stage interaction using a Wald test with Kenward-Roger degrees of freedom [60, 61], and a Type I error rate of 0.05. Power was computed for the same hypothesis and model as planned for data analysis, using GLIMMPSE [27] version 3.0.0. The GLIMMPSE platform utilizes the Hotelling Lawley Trace test instead of the usual mixed model Wald test. Under many conditions, the Wald test coincides with the Hotelling Lawley Trace test, and therefore the power computations are equivalent [12]. Power computations assumed a sample size of 500 adults and 500 children, with no more than 10% loss to follow-up. The recruitment feasibility and the loss-to-follow up rate for this population were previously studied by our team [4]. Means, standard deviations, and correlations were assumed to be as shown in Tables 1 and 2, and were obtained from data published by Olsen et al. [56], and from a study by the investigators in the same population [4]. The sensitivity of the power calculations to misspecification of means and standard deviations is shown in Fig. 2. The power appears to be sufficient even if the inputs are slightly mis-specified. Under all the assumptions made in the paragraph, the proposed study is predicted to have power of at least 0.82.

We have included a step-by-step guide (Additional files 1 & 2) for performing the power analysis shown in the manuscript in GLIMMPSE [27]. The power analysis can be completely replicated using Additional file 1. In addition, we’ve included step-by-step screen shots for the software (Additional file 2), showing how to provide the inputs, and how to describe the design and the hypothesis.

## Discussion

Power analysis is an important component of designing a replicable study. However, well-defined approaches to power analysis are seldom taught to scientists. The environmental health sciences literature has few descriptions of approaches for power and sample size analysis for longitudinal mixed models. Further, power and sample software can be challenging to use. This manuscript attempts to fill the gap.

Even when approaches for power and sample size analysis are well-understood, choosing reasonable inputs can be difficult. Power analysis may be challenging because it requires speculation about unknowns. Before a study starts, a researcher will not know what means, variances and correlations to expect, although they may understand what differences in health outcomes will be important to peoples’ wellbeing. Even with pilot studies, it is not possible to predict the means or standard deviations that will be observed in a planned longitudinal study. This leaves the researcher in a position where they must guess at the optimal inputs, and then justify reasoning to themselves and their peers. A particular challenge is finding an appropriate covariance matrix. In data analysis, Gurka et al. [33] suggested that using an unstructured covariance matrix is the approach least likely to inflate Type I error rate. For power analysis, specifying an appropriate unstructured matrix may be challenging without preliminary data. The important thing is to clearly state which inputs are guesses and which inputs are derived from published or unpublished data.

This manuscript seeks to demystify the choice of inputs for power analysis. We offer suggestions for how to choose the best inputs, and how to document the limitations of the chosen inputs. We show how inputs for longitudinal studies may be inferred from pilot data or extracted from a systematic literature search. We show how to account for the complexities that arise with power and sample size analysis for longitudinal studies. With a step-by-step check list and the appropriate software tools, power analysis should not be overwhelming.

Power analysis inputs do not need to be perfect. The goal is that a researcher chooses inputs that make the most sense for the given population and study design. With this comes the responsibility of documenting the extent to which inputs are from a different population, a similar, but not exactly the same exposure, or a related, but not identical outcome. For example, cross-sectional pilot data might be available for the target study population, but might not provide insight about how repeated measurements will differ over time. A systematic literature search may provide information about measurements of health effects over time, but may not include the same mixture of chemical exposures observed for the planned study.

Given that inputs for power analysis may be imperfect, a rigorous scientist may want to see how slight misspecifications in inputs will affect a sample size calculation. We show how to use sensitivity analyses to assess the effect of inputs on the calculations. Scientists can compare power calculations performed with their best guess of inputs to power calculations with smaller mean differences and/or larger standard deviations. Scientists can then argue that even if the means, variance and correlations differ from what was specified in the power analysis, the resulting sample size will still provide enough power. On the other hand, knowing that a study may not have enough participants ahead of time can spur redesign, increased recruitment efforts, or expansion of the recruitment catchment area.

For many study designs, power and sample size analysis may be an iterative process of looking at inputs, checking the sample size required, and redesigning the study. Being able to document and archive power analyses is a key tenet of reproducible research. The GLIMMPSE platform, used for the power and sample size analyses in this paper, provides the ability to save all of the inputs and the study design. In this way, researchers can revisit the power analysis, and ask the question “What if this input changed?”

The GLIMMPSE platform uses a validated point-and-click approach for power and sample size analysis. The software provides guidance for power analysis by prompting the user for outcomes, predictors, repeated measurements, clustering, covariates, and study hypotheses. Hypothesis test options include tests for main effects, linear trends, interactions, nonconstant polynomials, and difference scores. Once the structure of the model is defined, GLIMMPSE then prompts the user for the required inputs. For longitudinal models, the software provides assistance in specifying a covariance structure that accounts for correlation between repeated measurements across time.

In this manuscript, we present an approach for power analysis for predicting declines in blood concentrations of persistent environmental chemicals. The approach has several strengths. Contributions include a discussion of the use of linear models for persistent chemicals, the utility of aligning power and data analysis, an explanation about selecting inputs from closely related literature, and a demonstration of how to describe a power analysis. Researchers may find the screenshots of our power calculation in GLIMMPSE (Additional file 2) useful as guides for their own power analysis.

Our approach also includes several weaknesses. This manuscript provides an example power analysis for PFAS, a persistent chemical. The same modeling approach, and a similar power analysis may not be reasonable for non-persistent chemicals. Linear mixed models assume that the outcome is continuous, that the errors are normally distributed, and that the concentration of the chemical is approximately linear as a function of the predictor values across time. The rapid metabolism of non-persistent chemicals leads to high within-person variability. The problem is further complicated if people have repeated exposure to the chemicals. Rapid metabolism and repeated exposure make it difficult to model how internal concentrations change between measurements. It is unlikely that concentrations of non-persistent chemicals follow a linear or polynomial curve across time. This feature violates the assumptions of the linear mixed model, making the model an inappropriate choice for repeated measurements of non-persistent chemicals.

New modeling methods for complex outcomes, such as non-persistent chemicals, outpace methods for power analysis. Better methods for modeling non-persistent chemicals are continually appearing in the field of environmental science. Each new method presents a new challenge for power analysis. Currently, power analysis tools are not available for techniques including Bayesian kernel machine regression, lasso regression, or weighted quantile sum regression.

For analytic approaches which have no power and sample size methodology, simulation is a common approach for aligning the power analysis with the planned analysis. Custom simulations require custom-built code, which is complicated to write, and difficult to check. For validation, simulations must undergo unit and overall testing. Achieving software industry standards for correctness is expensive in terms of time, effort and skill. To meet scientific research standards for transparency, one must post the code as open source so that others may check it.

## Conclusion

All investigators have an ethical responsibility to appropriately power environmental health sciences research. Although there are many unknowns, a consistent approach to power analysis allows researchers to select sample sizes as accurately as possible. A strong power analysis approach includes clearly defining a testable hypothesis, defining complementary methods for modeling and power analysis, obtaining power analysis inputs from pilot data or published resources, and conducting the power analysis with validated software. It is our hope that this tutorial and checklist will assist environmental health scientists in confidently planning and conducting power analyses for longitudinal studies of continuous outcomes.

## Supplementary Information

**Additional file 1.** This .txt file provides inputs for GLIMMPSE version 3.0.0 software.

**Additional file 2.** This .pdf file provides step-by-step screenshots for conducting our power analysis in the GLIMMPSE version 3.0.0 software.

## Data Availability

All data generated or analyzed during this study are included in this published article [and its supplementary information files].

## Abbreviations

AFFF: aqueous film-forming foams
LEAR: linear exponential autoregressive
NIH: National Institute of Health
PFAS: per- and polyfluoroalkyl substances
PFHxS: perfluorohexane sulfonate
PFOS: perfluorooctane sulfonate
PFOA: perfluorooctanoate
USEPA: United States Environmental Protection Agency.
