# Supplementary material for: Power and sample size analysis for longitudinal mixed models of health in populations exposed to environmental contaminants: a tutorial
Source: BMC Med Res Methodol. 2023 Jan 12;23:12. doi: 10.1186/s12874-022-01819-y (PMC9835314; doi:10.1186/s12874-022-01819-y)
Supplement: Supplementary file 2 — Additional file 2. This .pdf file provides step-by-step screenshots for conducting our power analysis in the GLIMMPSE version 3.0.0 software. [file 12874_2022_1819_MOESM2_ESM.pdf]

This document contains screen shots for replicating the power analysis example from our manuscript. The GLIMMPSE V3 software may be found at https://samplesizeshop.org.

- Simply click "Calculate Sample Size Now" to begin.

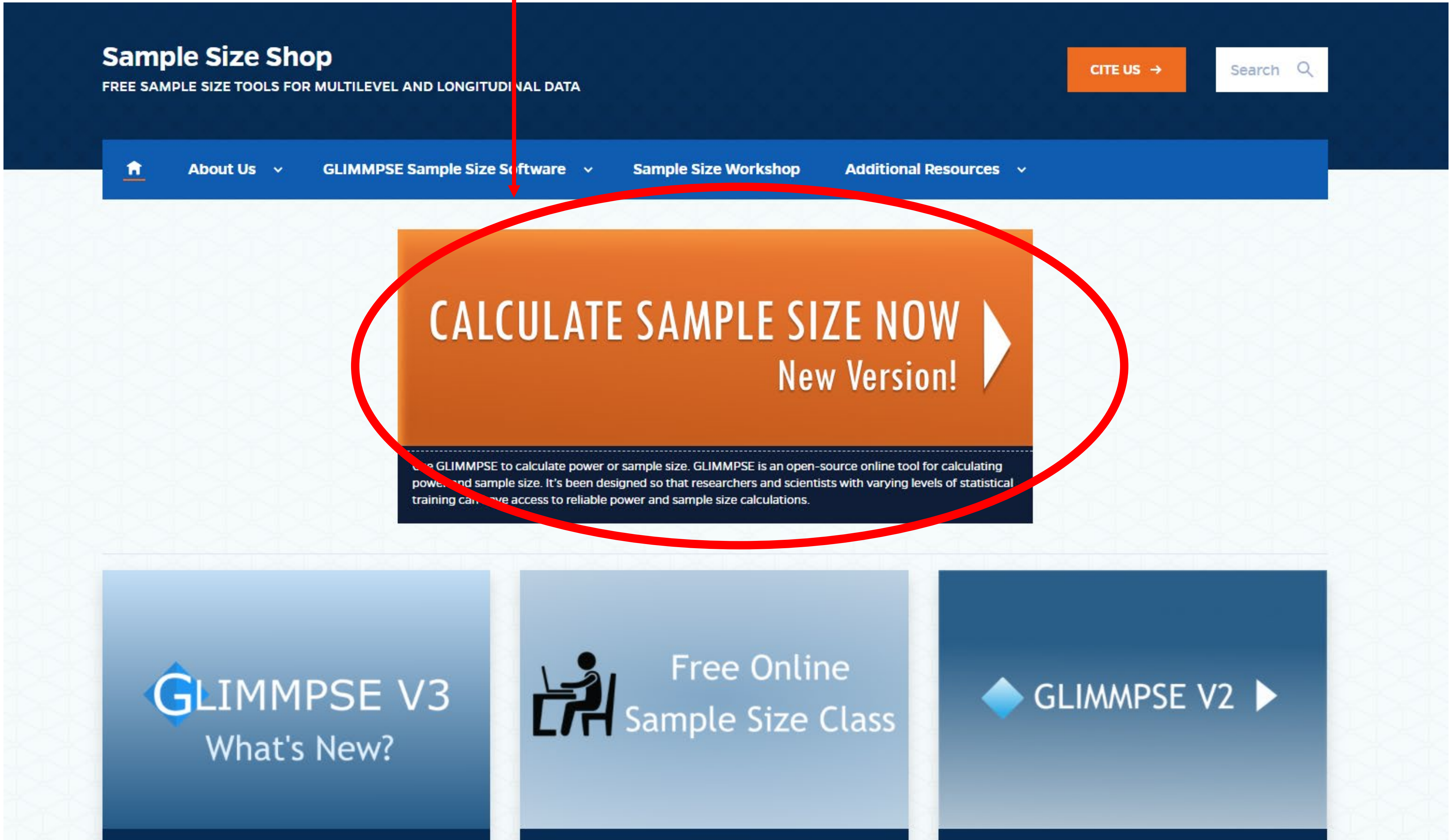

Begin your study design.

- Click the "New Study" button to start a new power and sample size analysis.
- Click the "Upload" button to upload a json file with a previous design that you have saved. To replicate the power analysis example from our manuscript, upload EnvironmentalPower01.json. This file may be found in supplemental information.

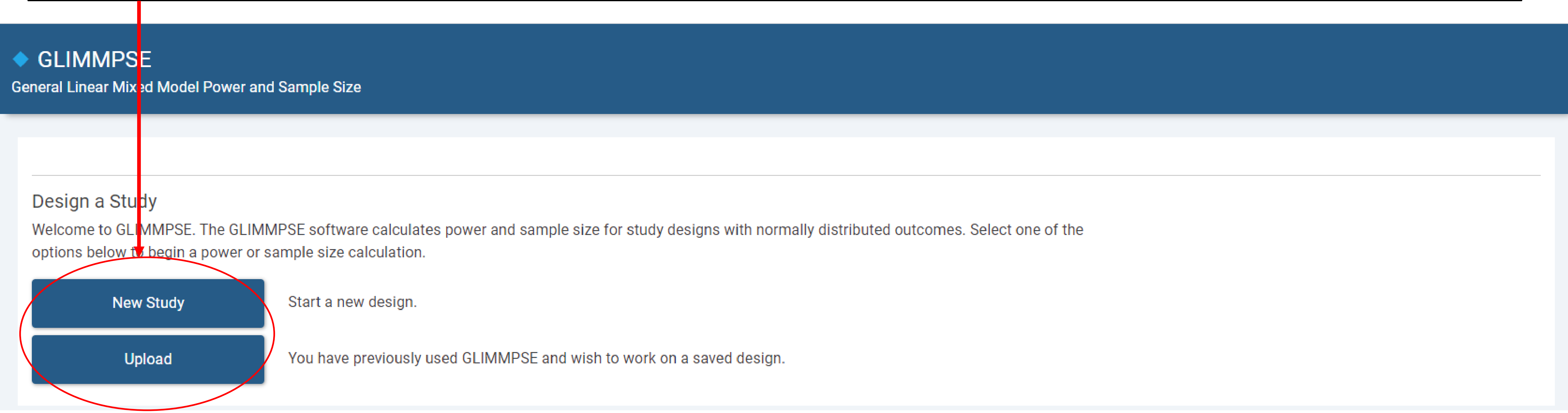

Indicate if you want to solve for power or sample size.

- Click “Power” to solve for power.
- Click “Sample Size” to solve for sample size.

- We chose power and navigated to the next page.

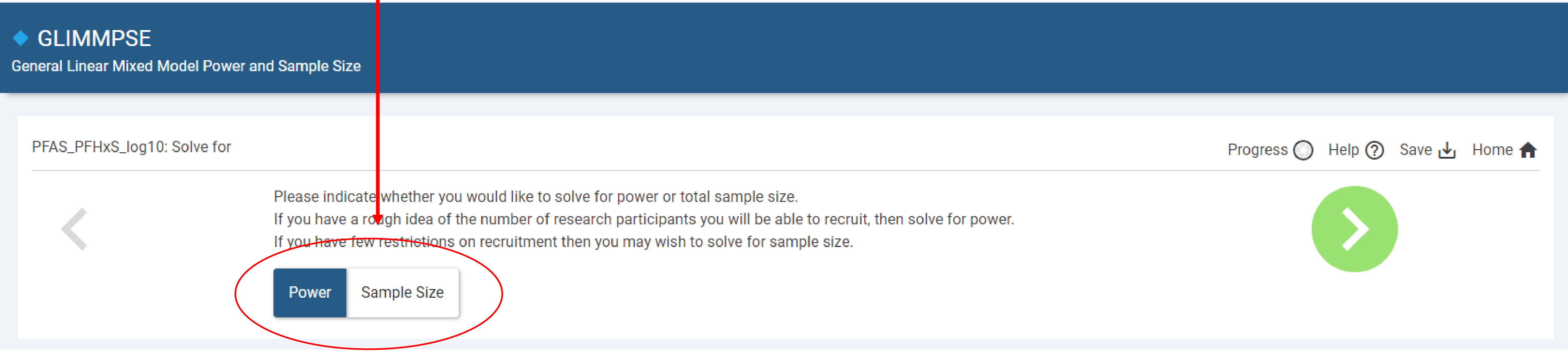

Choose the statistical test.

- If unsure, we recommend the Hotelling Lawley Trace test. Under many conditions, the mixed model Wald test coincides with the Hotelling Lawley Trace test for the multivariate model, and therefore the power computations are equivalent. For more information, please see Chi et al. 2019 (12).

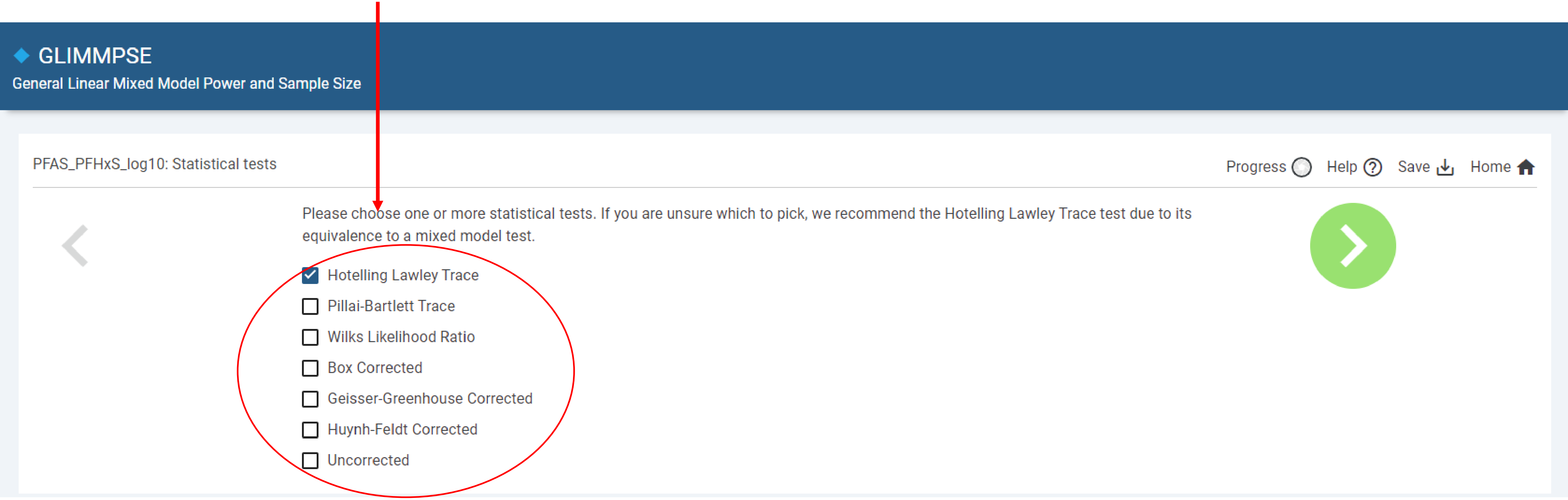

Choose the Type I error rate.

- We chose 0.05 and then clicked the plus sign to add the value to the design.

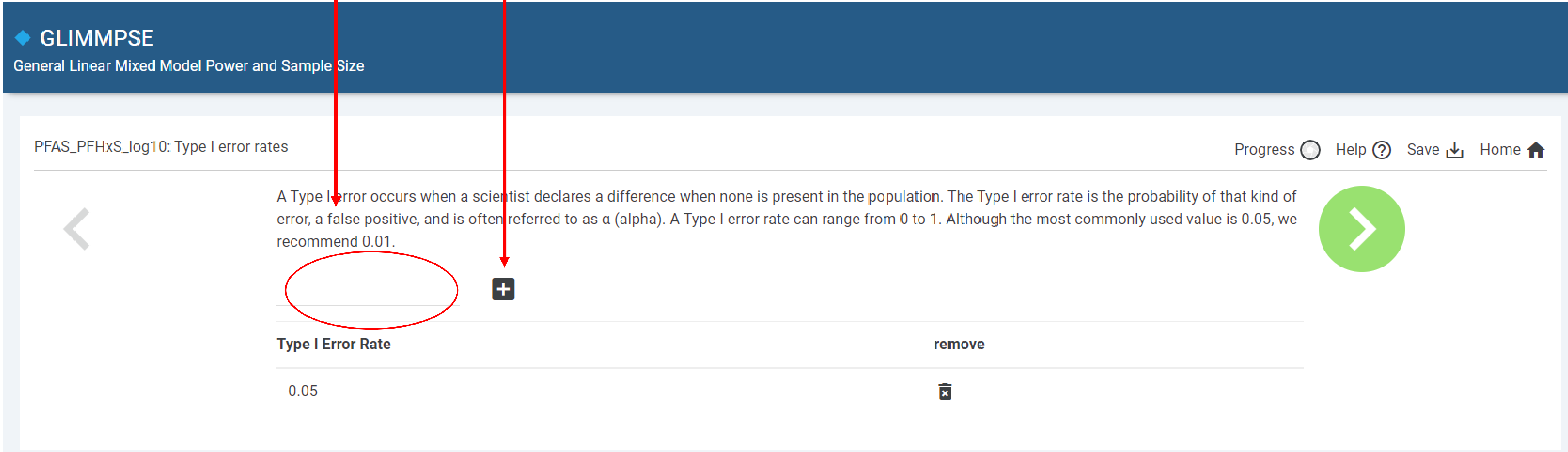

Enter your outcome.

- We entered PFHxS and clicked the plus sign to add it to our design.

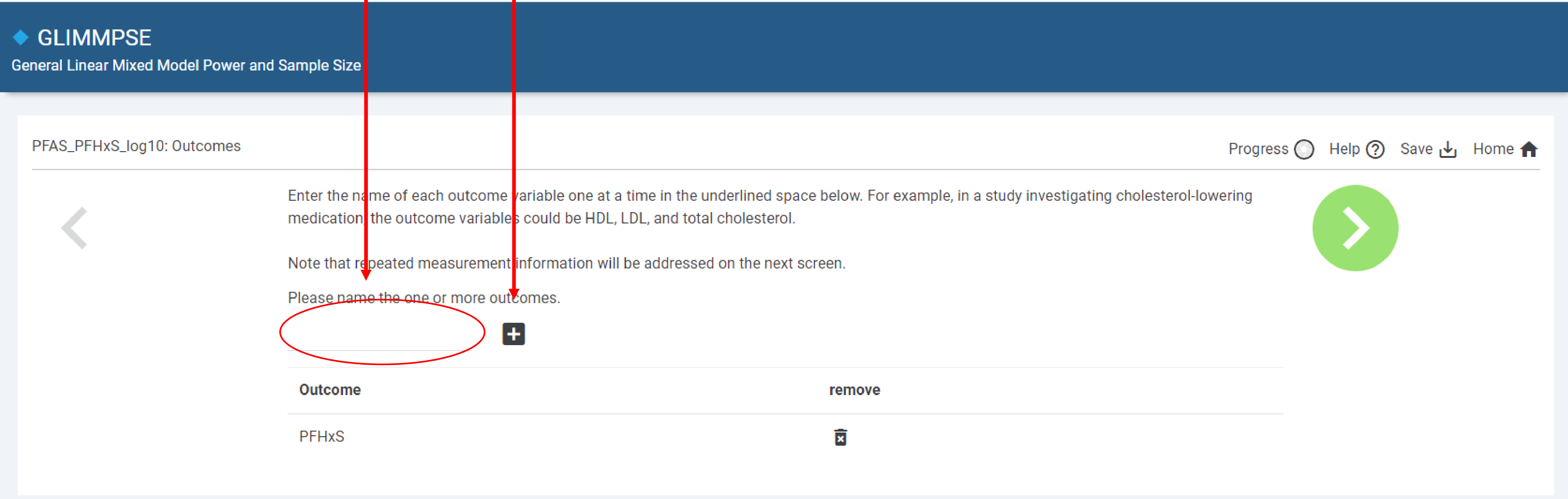

Click on “Define Repeated Measure” to add repeated measures to the design.

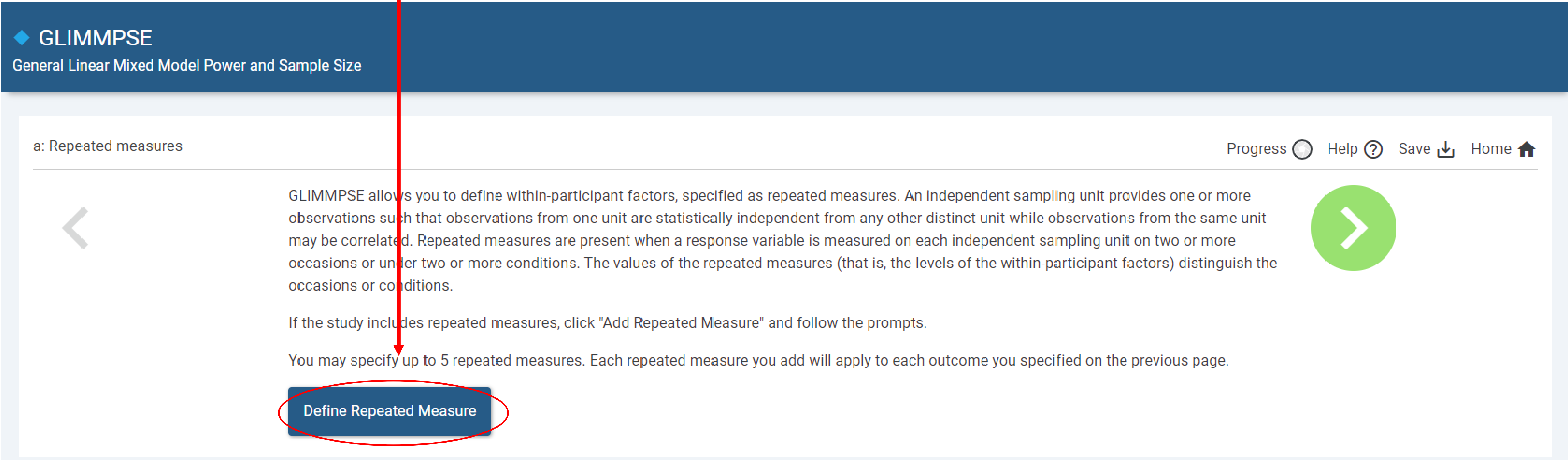

Define the dimension of your repeated measurements.

- We chose time and clicked “Next: Type”.

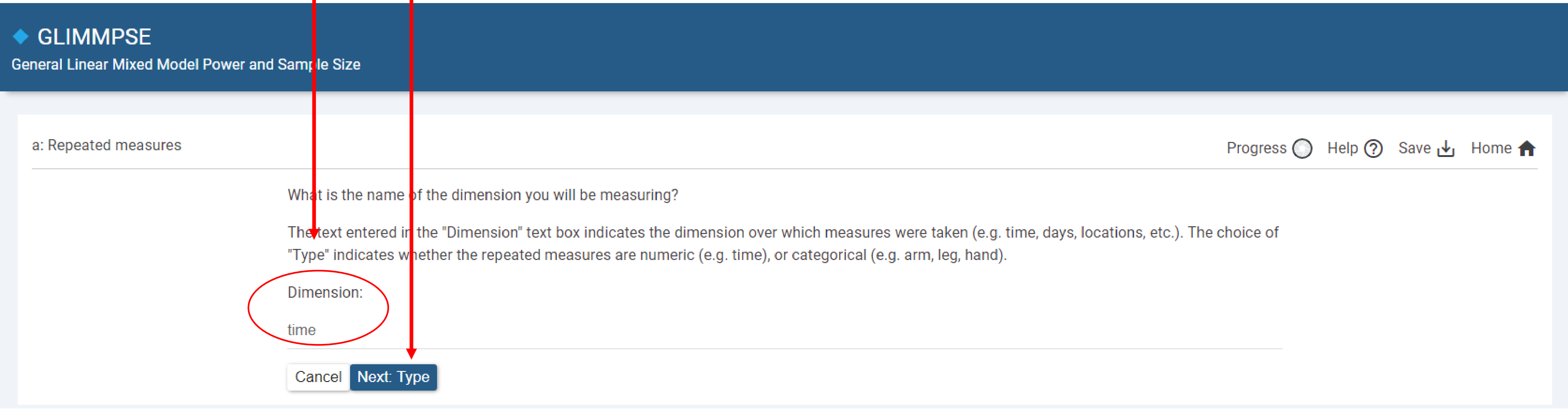

Choose the data type that best defines the dimension of your repeated measurements.

- We chose "Numeric" and clicked on "Next: No. Measurements".

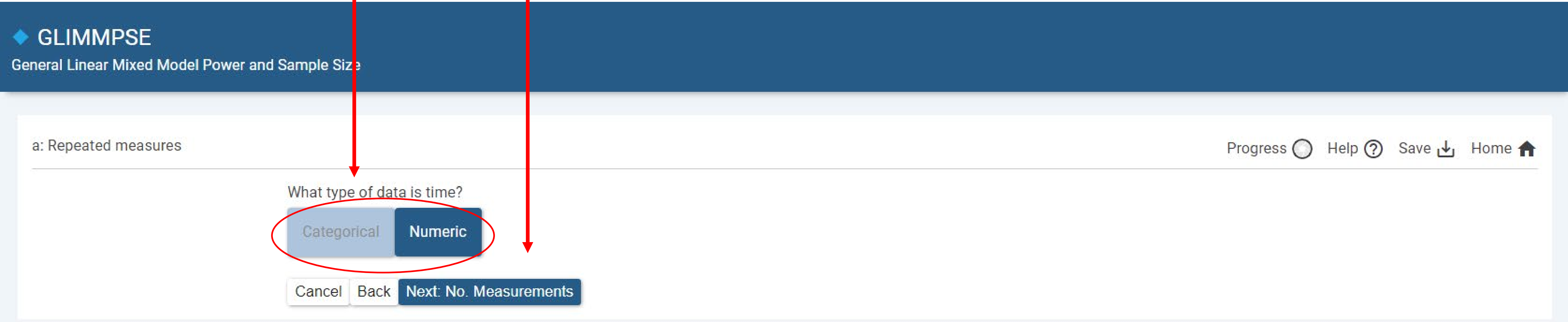

Choose the number of repeated measurements.

- We chose “3” and clicked on “Next: Spacing”.

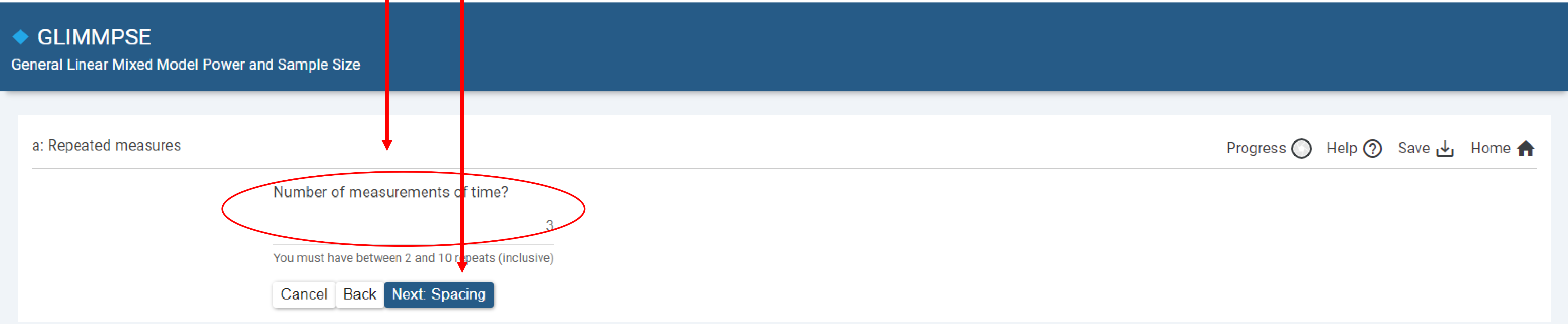

Choose the spacing of the repeated values.

- Click “Set values myself” to choose your own values.
- Click “Select values by series” to choose values using a series of numbers.

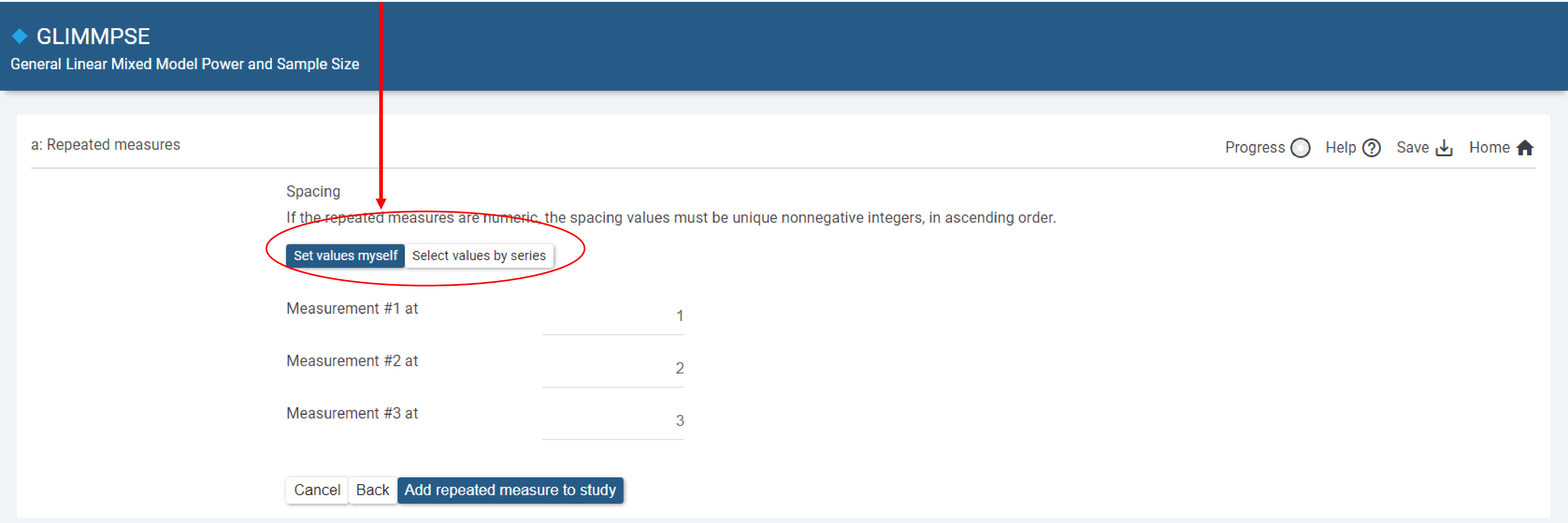

- We chose “Set values myself” and entered 1, 2, and 3.

- We then clicked “Add repeated measure to study”.

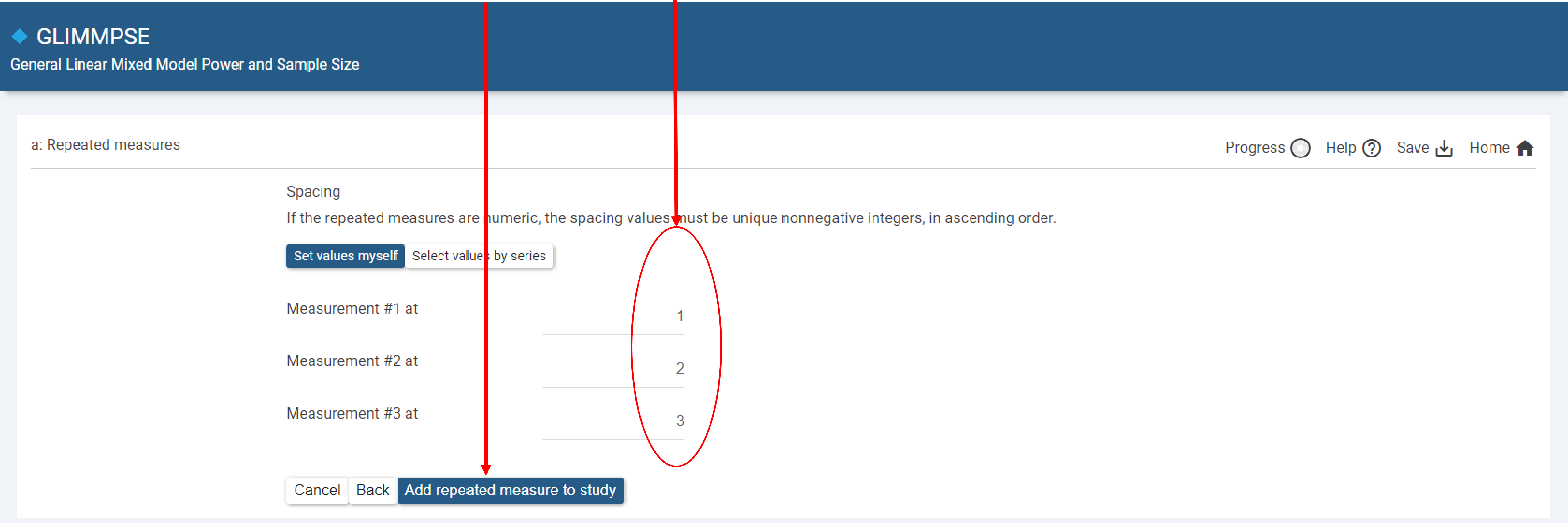

Navigate to the next page.

◆ GLIMMPSE

General Linear Mixed Model Power and Sample Size

PFAS_PFHxS_log10: Repeated measures

Progress Help Save Home

GLIMMPSE allows you to define within-participant factors, specified as repeated measures. An independent sampling unit provides one or more observations such that observations from one unit are statistically independent from any other distinct unit while observations from the same unit may be correlated. Repeated measures are present when a response variable is measured on each independent sampling unit on two or more occasions or under two or more conditions. The values of the repeated measures (that is, the levels of the within-participant factors) distinguish the occasions or conditions.

If the study includes repeated measures, click "Add Repeated Measure" and follow the prompts.

You may specify up to 5 repeated measures. Each repeated measure you add will apply to each outcome you specified on the previous page.

Define Repeated Measure

Repeated Measure Dimension	Type	Measurements	Edit	Remove
time	Numeric	["1", "2", "3"]		

Account for data clustering in the design.

- We did not add clustering and navigated to the next page.

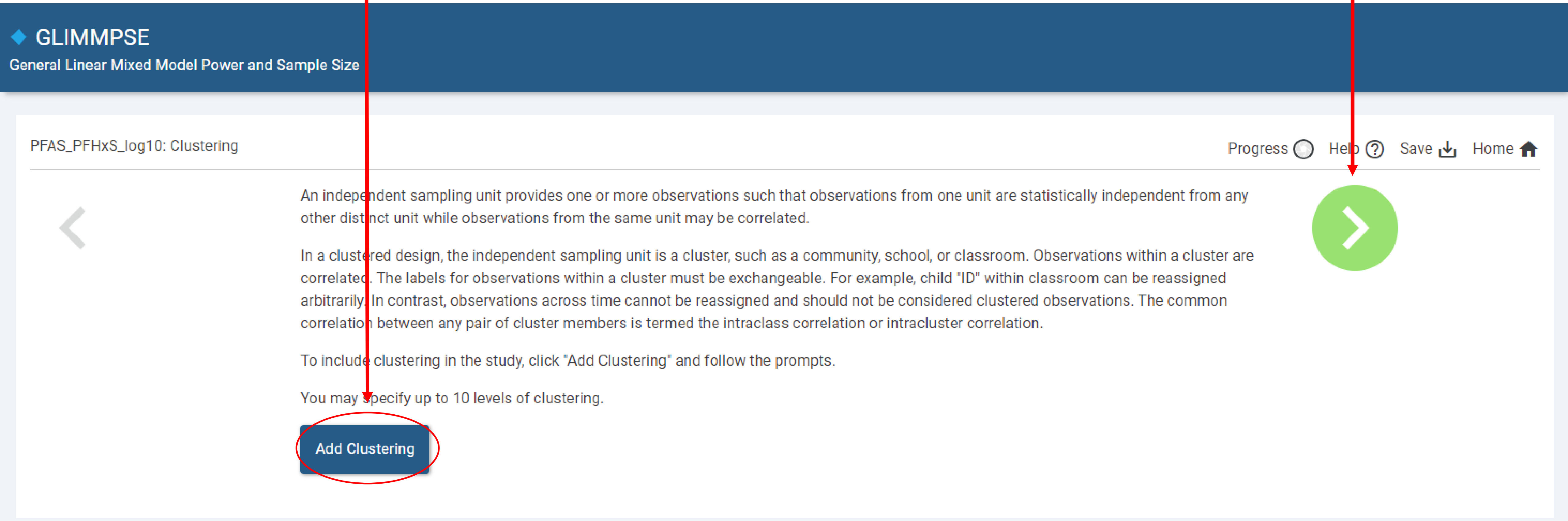

Click on “Define Fixed Predictor” to add your main predictor to the design.

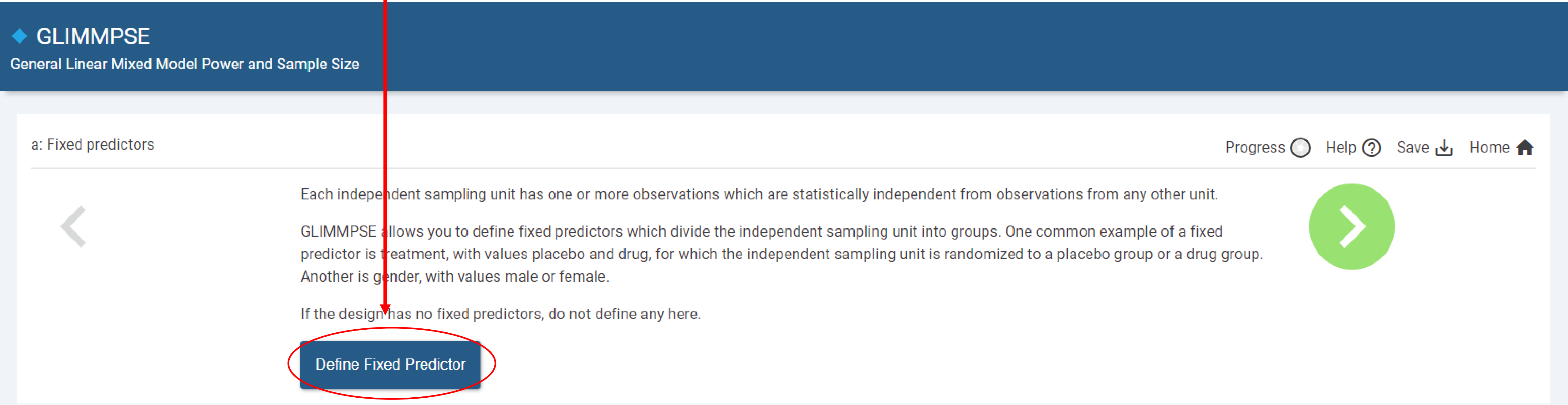

Enter your main predictor.

- We entered Adult and clicked on "Next: Data Type".

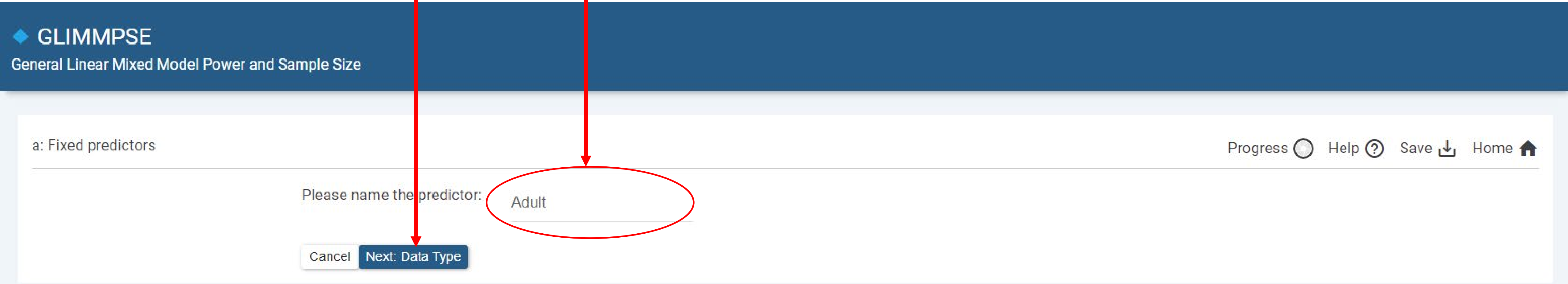

Choose the data type that best describes your predictor.

- Click “Nominal” if your predictor is categorical.
- Click “Continuous” if your predictor is continuous.

- We chose “Nominal” and clicked on “Next: Groups”.

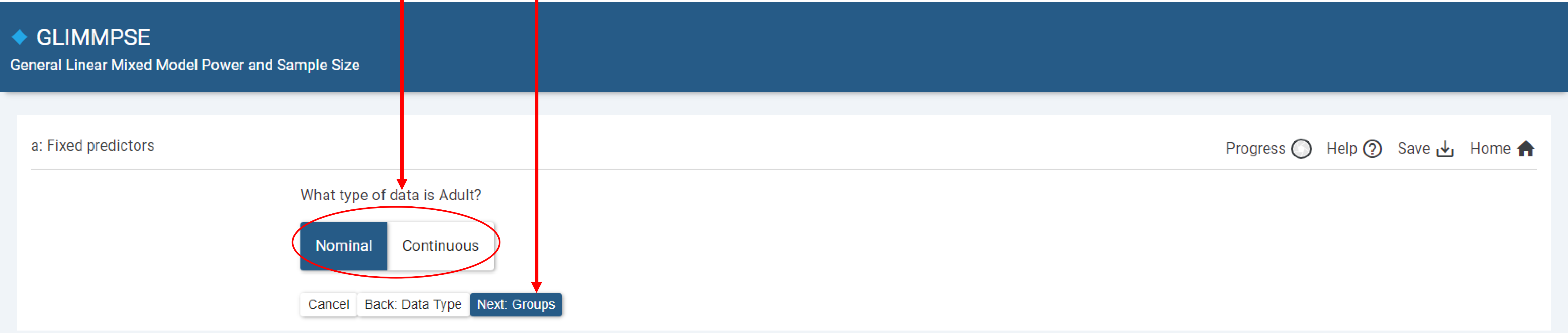

Name the categories of your nominal predictor.

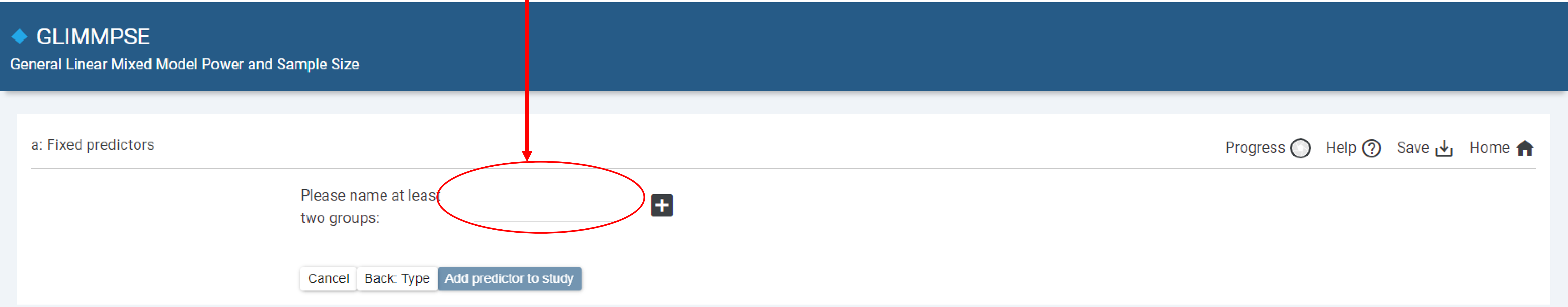

- We entered “Child” and clicked on the plus sign.
- We then entered “Adult” and clicked on the plus sign.

- We then clicked on “Add predictor to study”.

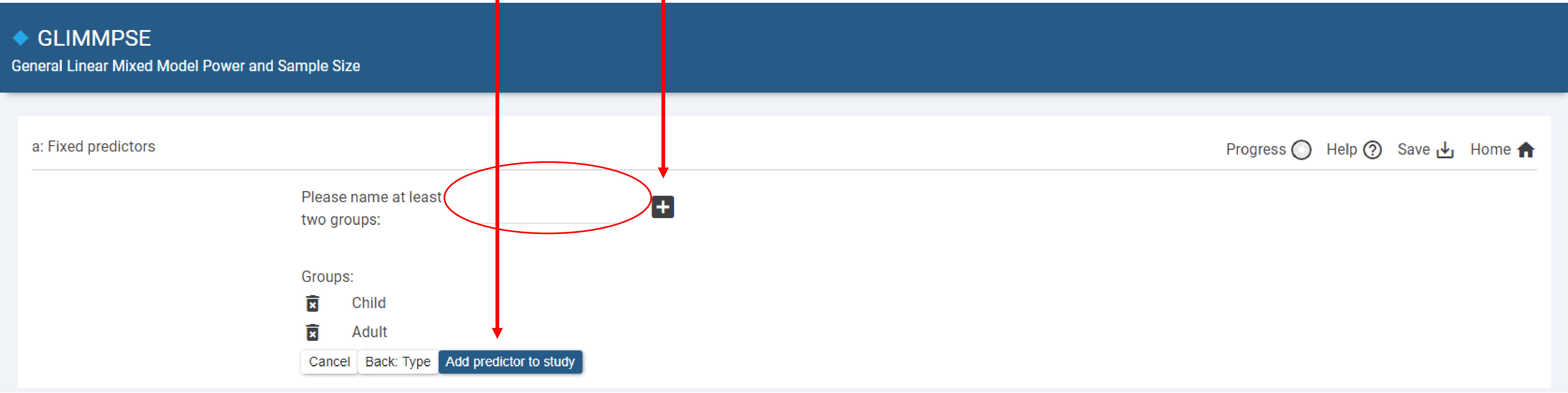

Navigate to the next page.

◆ GLIMMPSE

General Linear Mixed Model Power and Sample Size

PFAS_PFHxS_log10: Fixed predictors

Progress Help Save Home

Each independent sampling unit has one or more observations which are statistically independent from observations from any other unit.

GLIMMPSE allows you to define fixed predictors which divide the independent sampling unit into groups. One common example of a fixed predictor is treatment, with values placebo and drug, for which the independent sampling unit is randomized to a placebo group or a drug group. Another is gender, with values male or female.

If the design has no fixed predictors, do not define any here.

Define Fixed Predictor

Fixed Predictors

Name	Type	Units	Groups	Remove	Edit
Adult	NOMINAL		["Child", "Adult"]		

Add a continuous covariate to the design.

- Click on “No Gaussian Covariate” if you do not want to add a continuous control variable.
- Click on “Gaussian Covariate” if you do want to add a continuous control variable.

- We chose “No Gaussian Covariate” and navigated to the next page.

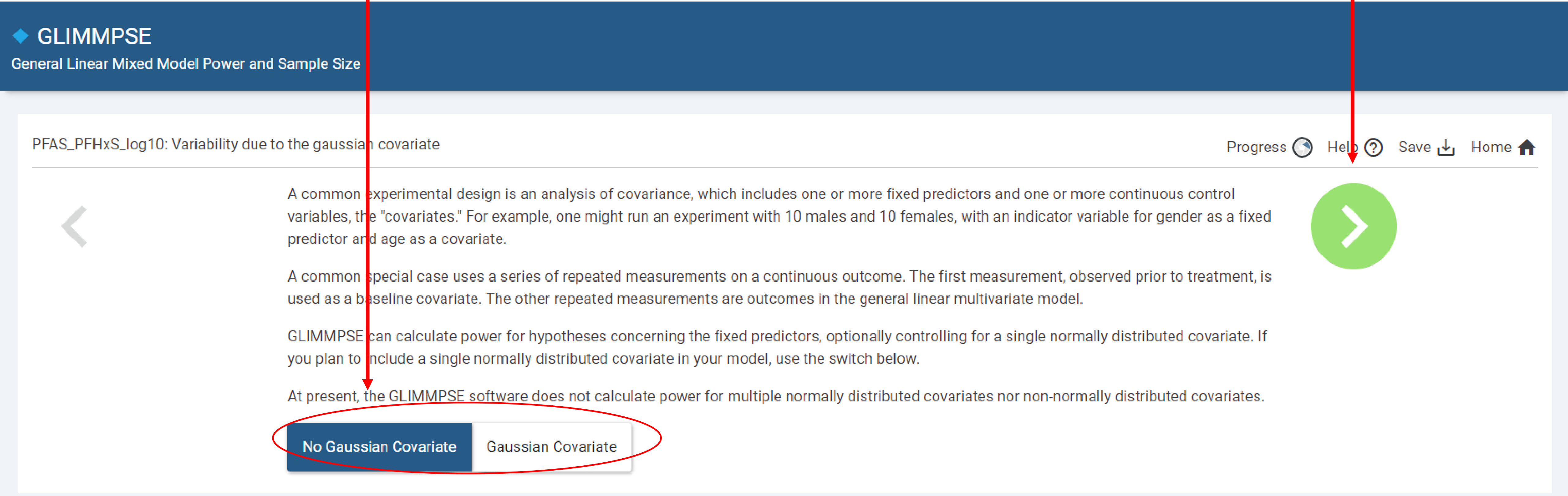

Choose a hypothesis test.

- We chose to test the interaction between our predictor “Adult” and time.

GLIMMPSE

General Linear Mixed Model Power and Sample Size

PFAS_PFHxS_log10: Hypothesis choice

Progress Help Save Home

Each power or sample size calculation is based on selecting a specific study hypothesis. The options below show the hypotheses which are available for the current study design. Specify the hypothesis that represents your scientific question.

GLIMMPSE chooses sensible contrast matrices based on cell means coding. Should you wish to define your own contrast matrices, pick the highest order interaction and choose from the advanced options in the hypothesis components.

Select a hypothesis from the list.

	Effects Available for Consideration	Nature of Variation
(●)	Adult x time: Interaction	Between x Within
( )	time: Main Effect	Within
( )	Adult: Main Effect	Between
( )	Grand Mean	Between

Define the contrast between means.

- We clicked on “All mean differences zero”.

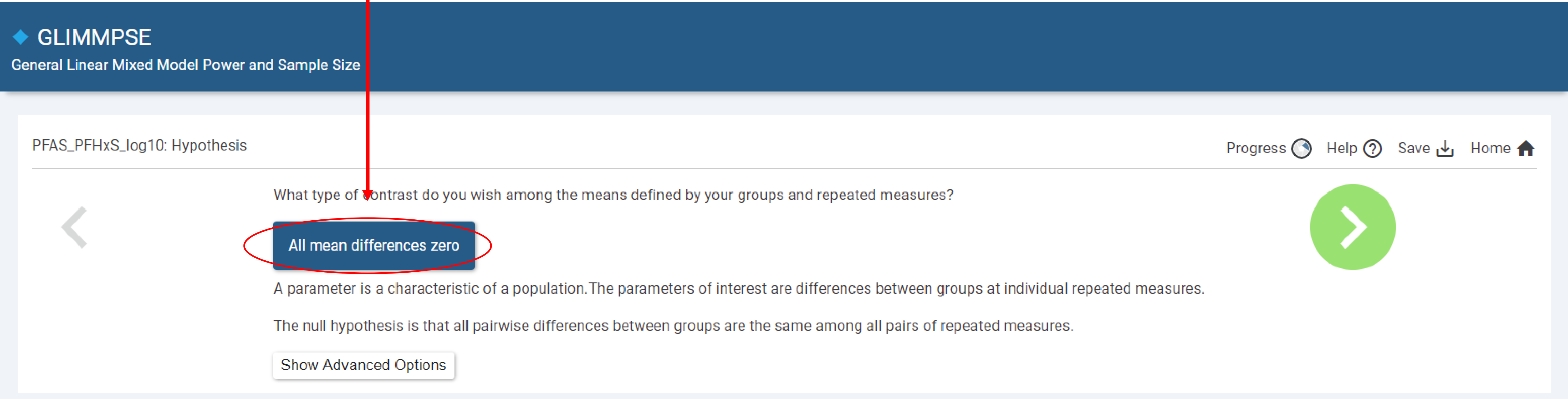

Select a value for the contrast comparison constant.

- We used the default of zero. If you’re not sure what to chose, use zero.

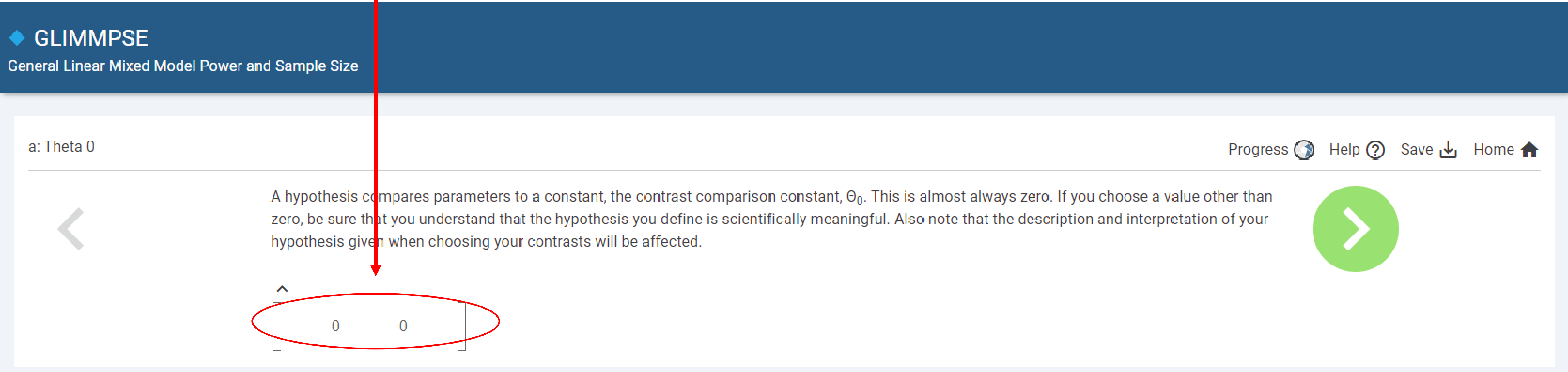

Enter the sample size for your smallest group.

- We entered “450” and clicked on the plus sign.
- While investigators planned for a sample size of 500, this number accounts for 10% attrition [i.e. 500 * (1 – 0.10)].

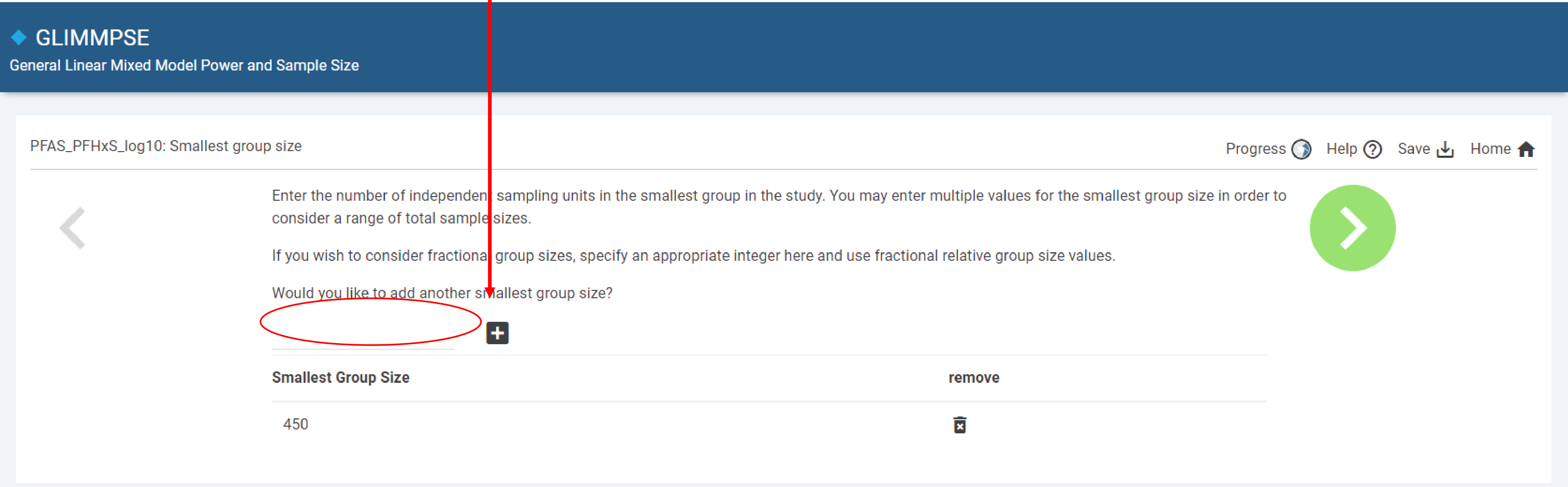

Enter your group size ratio.

- We have equal group sizes, so we set each group ratio to 1.

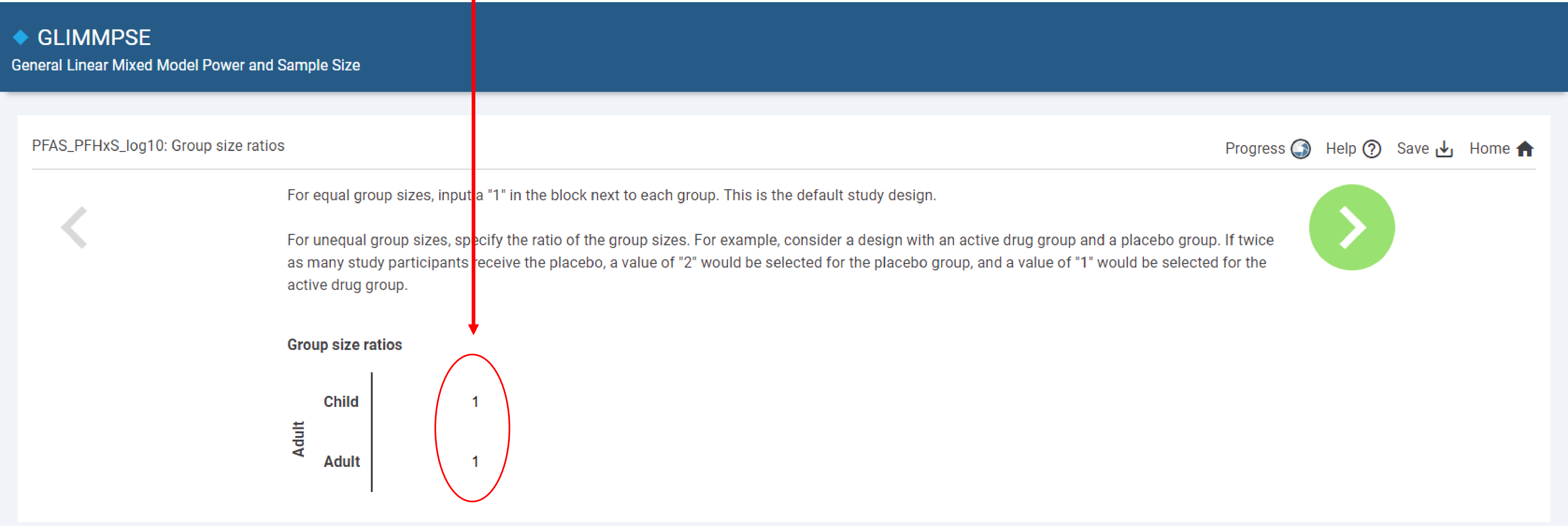

Enter mean values of your outcome across repeated measures and categories of your predictor.

GLIMMPSE
General Linear Mixed Model Power and Sample Size

PFAS_PFHxS_log10: Marginal means

Progress Help Save Home

The table below shows the mean values for outcome **PFHxS** within each group in the study. Each group is represented by a row in the table, and each repeated measure dimension is represented by a column.

Enter the mean values you expect to observe for outcome **PFHxS** within each group. The table should contain at least one value that is non-zero. Also, at least two groups should have means which differ by a scientifically meaningful amount.

Expected mean values, per group, for *PFHxS*

Adult	time: 1	2	3
Child	0.66	0.58	0.51
Adult	1.05	1.01	0.96

Set blank values to   value

Add scale factors for your means.

- A factor of 1 will compute power for the mean differences that you input. Make sure you do not delete this.
- A factor of 2 will compute power for mean differences twice as big.
- A factor of 0.5 will compute power for mean differences half as big.

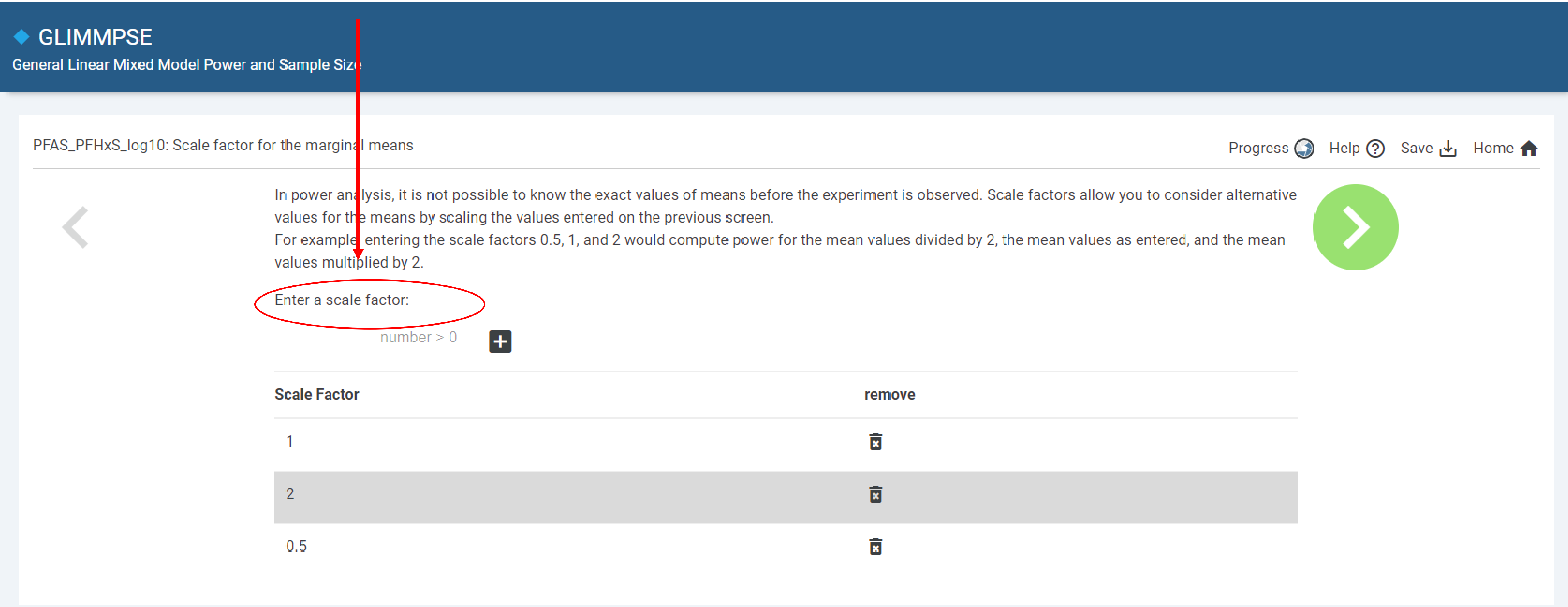

- We entered “2” and clicked on the plus sign.
- Then, we entered “0.5” and clicked the plus sign.

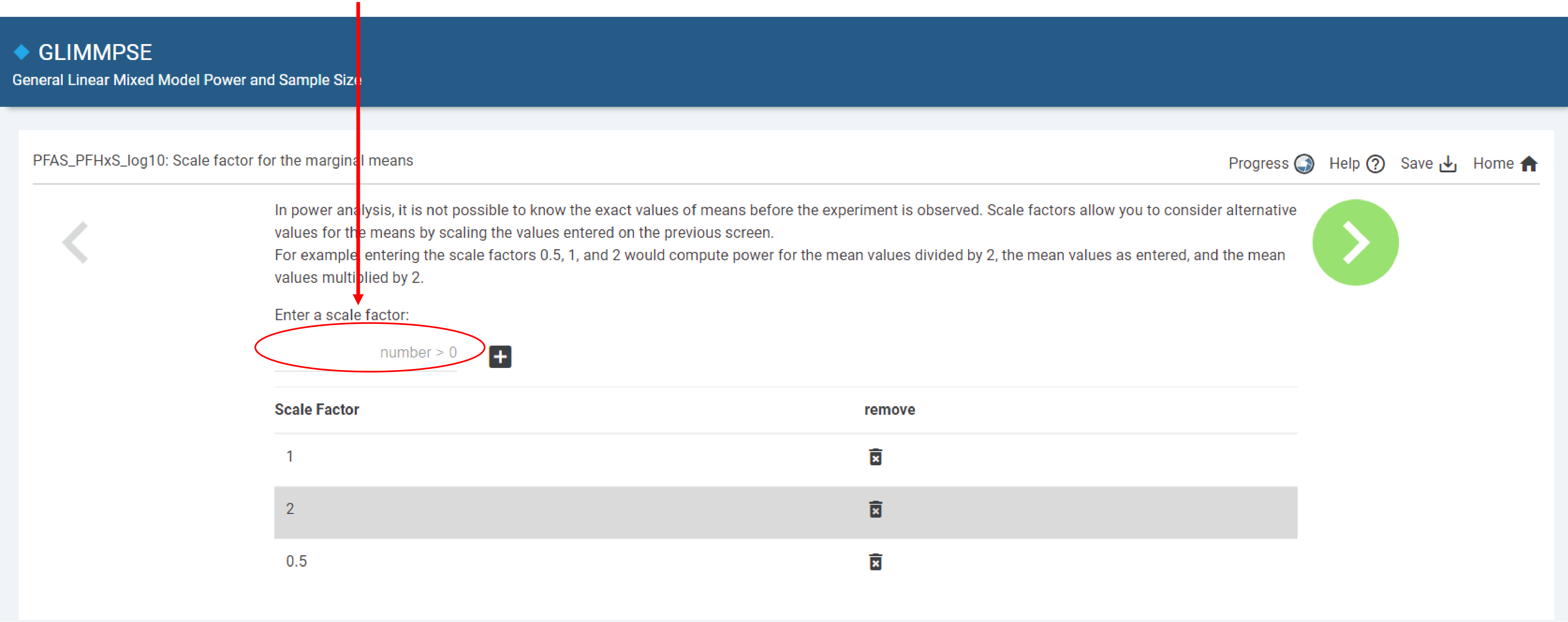

Enter the within-participant standard deviation for your outcome.

- We entered “0.48”.

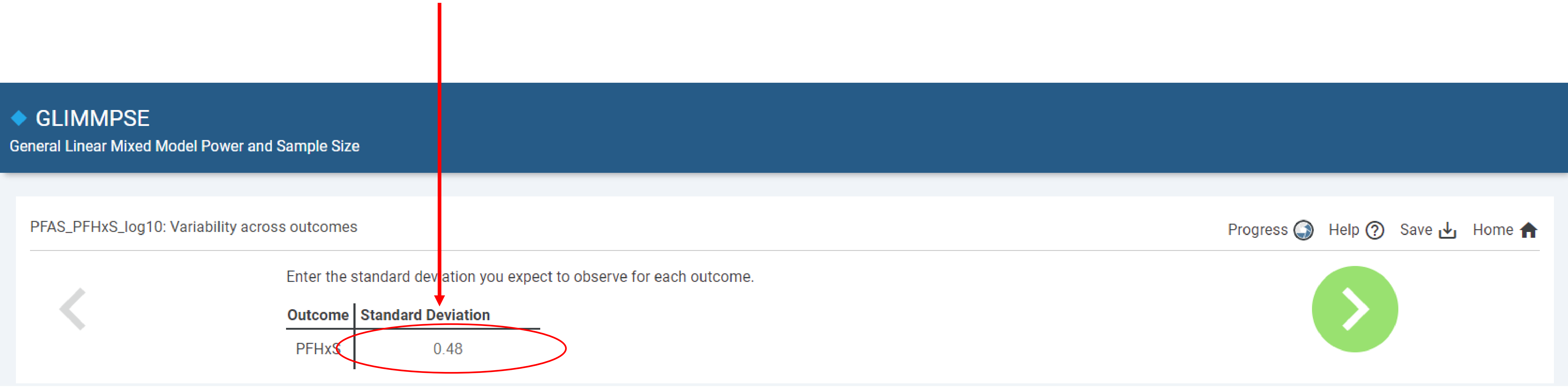

Enter repeated measure standard deviation ratios. This allows your standard deviation to change over time.

- We believed that our standard deviation would remain static over time, so we entered “1” for each time point.

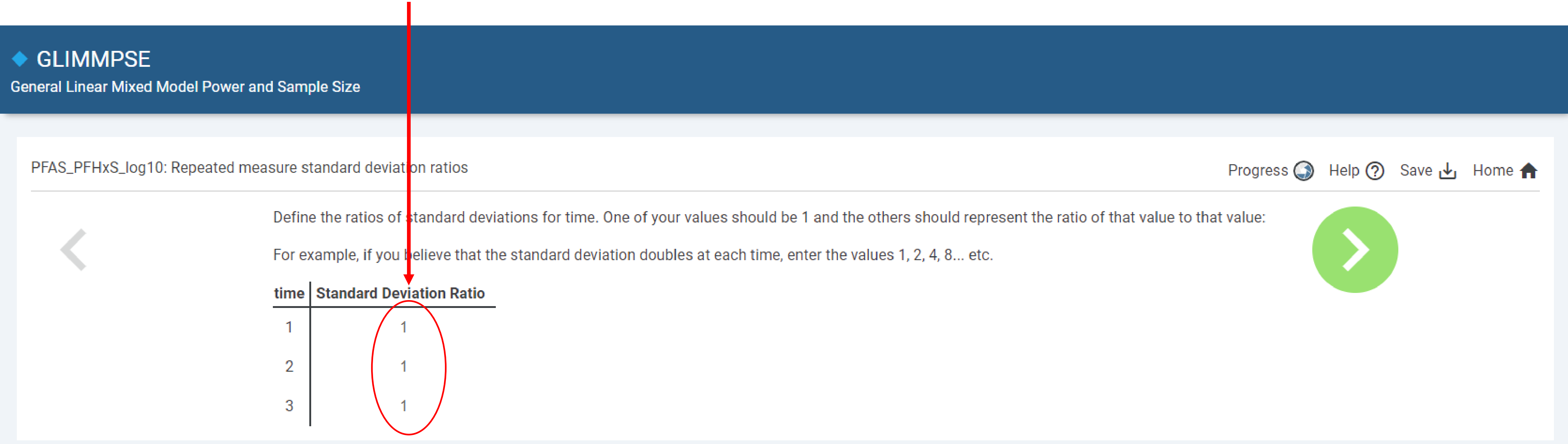

Choose either an unstructured or a linear exponent AR(1) (LEAR) covariance structure to model within participant correlation among repeated measurements.

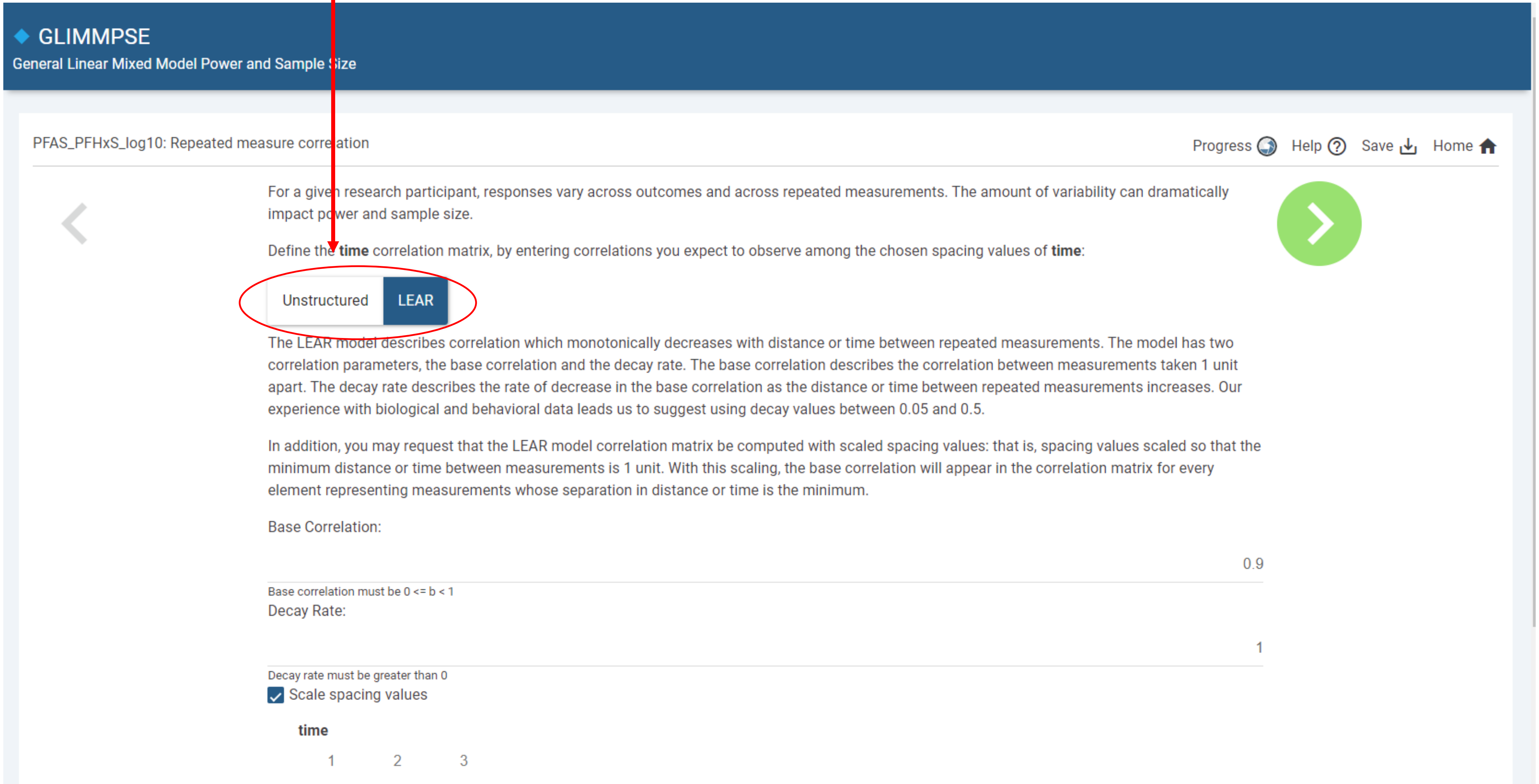

- We chose a LEAR covariance structure.

- We then entered a base correlation of “0.9” and a decay rate of “1”.

Unstructured | **LEAR**

The LEAR model describes correlation which monotonically decreases with distance or time between repeated measurements. The model has two correlation parameters, the base correlation and the decay rate. The base correlation describes the correlation between measurements taken 1 unit apart. The decay rate describes the rate of decrease in the base correlation as the distance or time between repeated measurements increases. Our experience with biological and behavioral data leads us to suggest using decay values between 0.05 and 0.5.

In addition, you may request that the LEAR model correlation matrix be computed with scaled spacing values: that is, spacing values scaled so that the minimum distance or time between measurements is 1 unit. With this scaling, the base correlation will appear in the correlation matrix for every element representing measurements whose separation in distance or time is the minimum.

Base Correlation: 0.9

Base correlation must be 0 <= b < 1

Decay Rate: 1

Decay rate must be greater than 0

☑ Scale spacing values

time

1	2	3
1	0.9	0.81
0.9	1	0.9
0.81	0.9	1

(each off-diagonal correlation must be between -1 and 1, exclusive)

Add scale factors for your standard deviation.

- A factor of 1 will compute power for the standard deviation that you input. Make sure you do not delete this.
- A factor of 2 will compute power for a standard deviation twice as big.
- A factor of 0.5 will compute power for a standard deviation half as big.

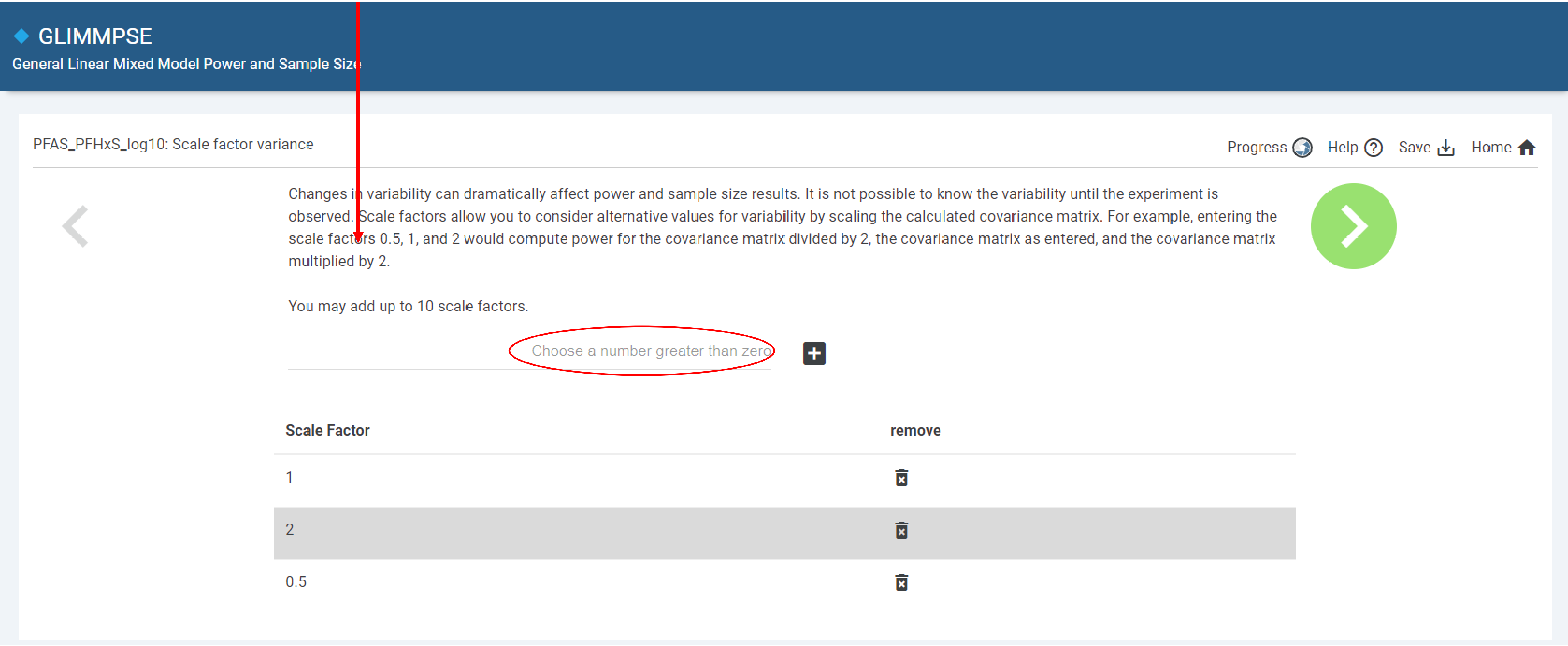

- We entered “2” and clicked on the plus sign.
- Then, we entered “0.5” and clicked the plus sign.

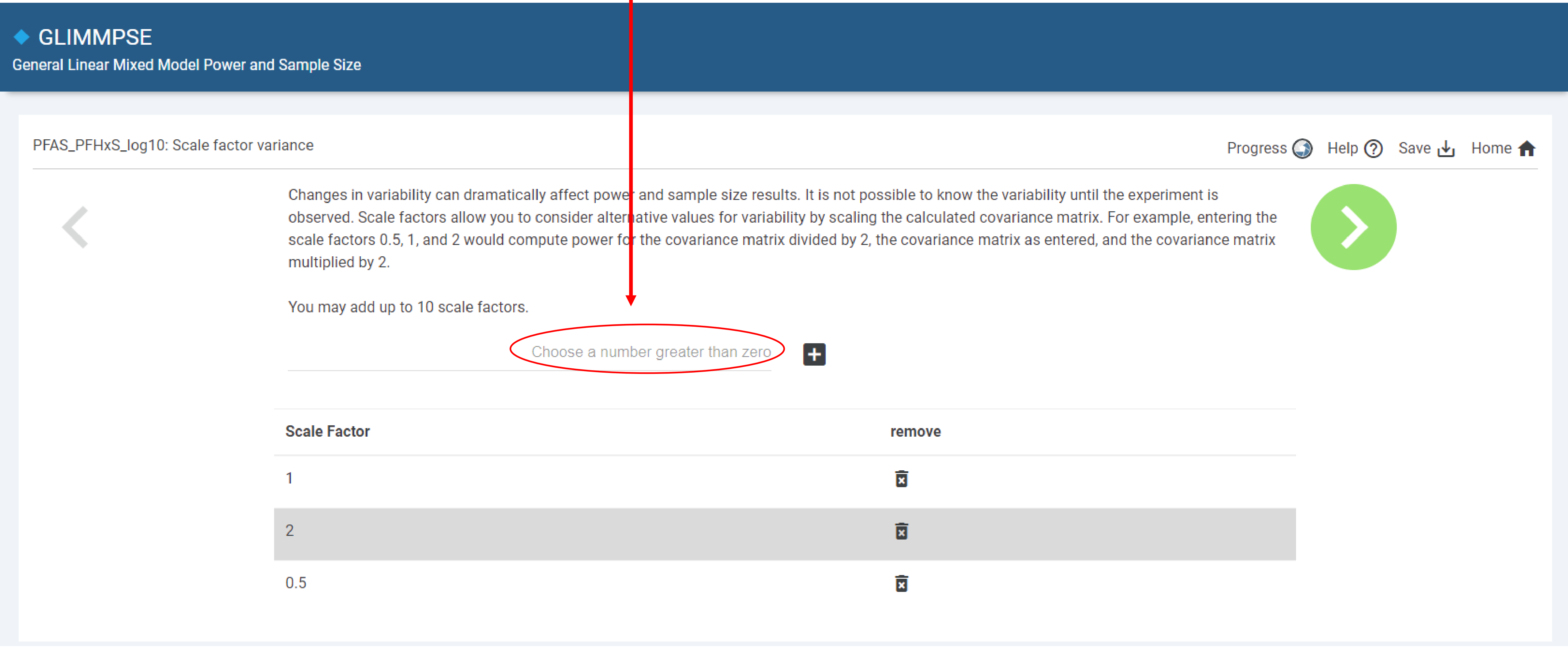

Compute confidence intervals for each computed power value.

- We did not compute confidence intervals, so we navigated to the next page.

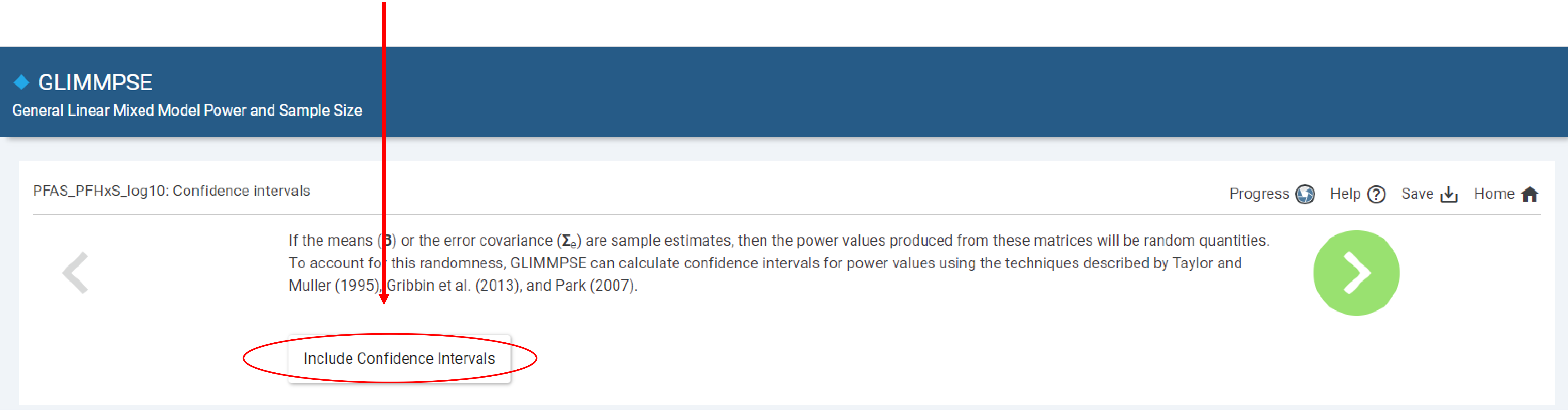

Click calculate to compute power.

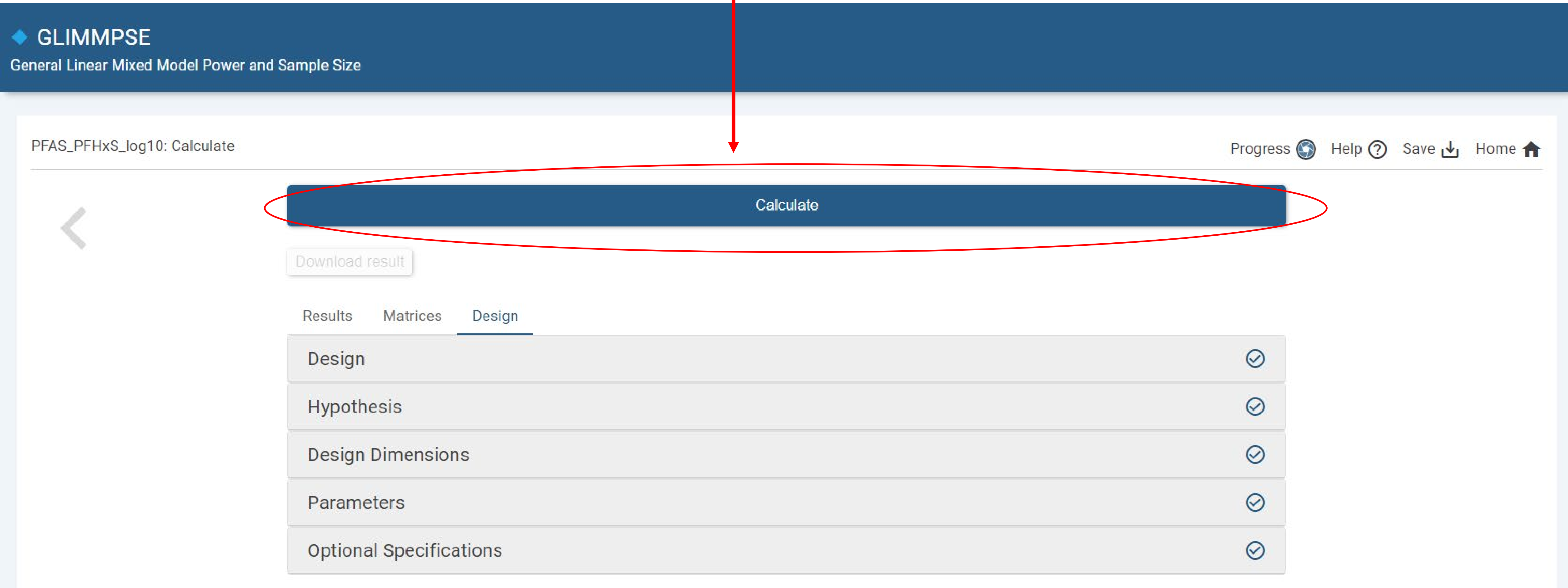

Table with power analysis results.

◆ GLIMMPSE

General Linear Mixed Model Power and Sample Size

PFAS_PFHxS_log10: Calculate

Progress Help Save Home

Calculate

Download result

Results Matrices Design

Power	Total Sample Size	Means Scale Factor	Variability Scale Factor	Test	Power Method	Type I Error Rate
0.821	900	1	1	Hotelling Lawley Trace	conditional	0.05
0.510	900	1	2	Hotelling Lawley Trace	conditional	0.05
0.986	900	1	0.5	Hotelling Lawley Trace	conditional	0.05
1.00	900	2	1	Hotelling Lawley Trace	conditional	0.05
0.986	900	2	2	Hotelling Lawley Trace	conditional	0.05
1.00	900	2	0.5	Hotelling Lawley Trace	conditional	0.05
0.277	900	0.5	1	Hotelling Lawley Trace	conditional	0.05
0.157	900	0.5	2	Hotelling Lawley Trace	conditional	0.05
0.510	900	0.5	0.5	Hotelling Lawley Trace	conditional	0.05

Power

GLIMMPSE

General Linear Mixed Model Power and Sample Size

PFAS_PFHxS_log10: Calculate

Progress Help Save Home

Calculate

Download result

Results Matrices Design

Power	Total Sample Size	Means Scale Factor	Variability Scale Factor	Test	Power Method	Type I Error Rate
0.821	900	1	1	Hotelling Lawley Trace	conditional	0.05
0.510	900	1	2	Hotelling Lawley Trace	conditional	0.05
0.986	900	1	0.5	Hotelling Lawley Trace	conditional	0.05
1.00	900	2	1	Hotelling Lawley Trace	conditional	0.05
0.986	900	2	2	Hotelling Lawley Trace	conditional	0.05
1.00	900	2	0.5	Hotelling Lawley Trace	conditional	0.05
0.277	900	0.5	1	Hotelling Lawley Trace	conditional	0.05
0.157	900	0.5	2	Hotelling Lawley Trace	conditional	0.05
0.510	900	0.5	0.5	Hotelling Lawley Trace	conditional	0.05

Sample size

GLIMMPSE

General Linear Mixed Model Power and Sample Size

PFAS_PFHxS_log10: Calculate

Progress Help Save Home

Calculate

Download result

Results Matrices Design

Power	Total Sample Size	Means Scale Factor	Variability Scale Factor	Test	Power Method	Type I Error Rate
0.821	900	1	1	Hotelling Lawley Trace	conditional	0.05
0.510	900	1	2	Hotelling Lawley Trace	conditional	0.05
0.986	900	1	0.5	Hotelling Lawley Trace	conditional	0.05
1.00	900	2	1	Hotelling Lawley Trace	conditional	0.05
0.986	900	2	2	Hotelling Lawley Trace	conditional	0.05
1.00	900	2	0.5	Hotelling Lawley Trace	conditional	0.05
0.277	900	0.5	1	Hotelling Lawley Trace	conditional	0.05
0.157	900	0.5	2	Hotelling Lawley Trace	conditional	0.05
0.510	900	0.5	0.5	Hotelling Lawley Trace	conditional	0.05

Scale factors of the mean difference

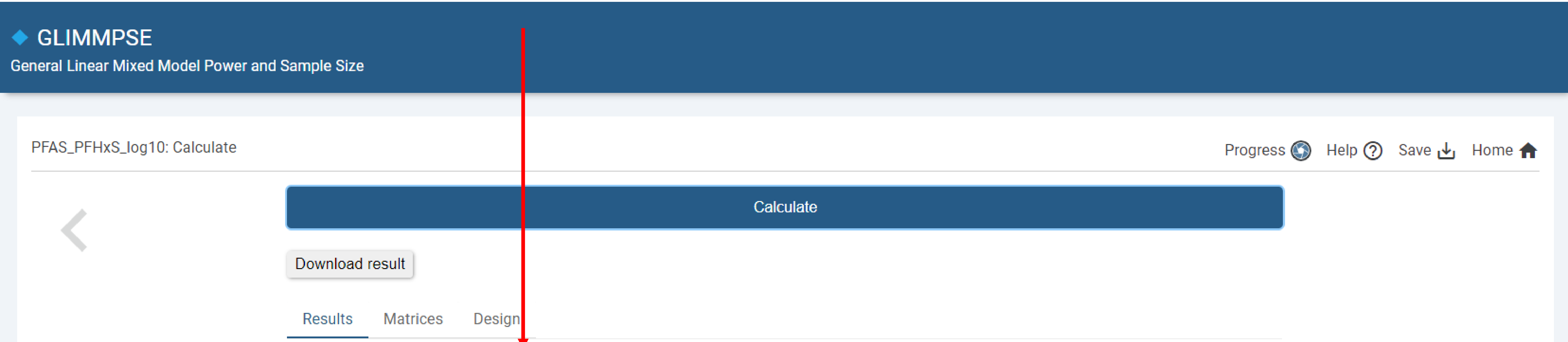

Power	Total Sample Size	Means Scale Factor	Variability Scale Factor	Test	Power Method	Type I Error Rate
0.821	900	1	1	Hotelling Lawley Trace	conditional	0.05
0.510	900	1	2	Hotelling Lawley Trace	conditional	0.05
0.986	900	1	0.5	Hotelling Lawley Trace	conditional	0.05
1.00	900	2	1	Hotelling Lawley Trace	conditional	0.05
0.986	900	2	2	Hotelling Lawley Trace	conditional	0.05
1.00	900	2	0.5	Hotelling Lawley Trace	conditional	0.05
0.277	900	0.5	1	Hotelling Lawley Trace	conditional	0.05
0.157	900	0.5	2	Hotelling Lawley Trace	conditional	0.05
0.510	900	0.5	0.5	Hotelling Lawley Trace	conditional	0.05

Scale factors of the standard deviation

GLIMMPSE

General Linear Mixed Model Power and Sample Size

PFAS_PFHxS_log10: Calculate

Progress Help Save Home

Calculate

Download result

Results Matrices Design

Power	Total Sample Size	Means Scale Factor	Variability Scale Factor	Test	Power Method	Type I Error Rate
0.821	900	1	1	Hotelling Lawley Trace	conditional	0.05
0.510	900	1	2	Hotelling Lawley Trace	conditional	0.05
0.986	900	1	0.5	Hotelling Lawley Trace	conditional	0.05
1.00	900	2	1	Hotelling Lawley Trace	conditional	0.05
0.986	900	2	2	Hotelling Lawley Trace	conditional	0.05
1.00	900	2	0.5	Hotelling Lawley Trace	conditional	0.05
0.277	900	0.5	1	Hotelling Lawley Trace	conditional	0.05
0.157	900	0.5	2	Hotelling Lawley Trace	conditional	0.05
0.510	900	0.5	0.5	Hotelling Lawley Trace	conditional	0.05

Download an excel file with your results

GLIMMPSE

General Linear Mixed Model Power and Sample Size

PFAS_PFHxS_log10: Calculate

Progress Help Save Home

Calculate

Download result

Results Matrices Design

Power	Total Sample Size	Means Scale Factor	Variability Scale Factor	Test	Power Method	Type I Error Rate
0.821	900	1	1	Hotelling Lawley Trace	conditional	0.05
0.510	900	1	2	Hotelling Lawley Trace	conditional	0.05
0.986	900	1	0.5	Hotelling Lawley Trace	conditional	0.05
1.00	900	2	1	Hotelling Lawley Trace	conditional	0.05
0.986	900	2	2	Hotelling Lawley Trace	conditional	0.05
1.00	900	2	0.5	Hotelling Lawley Trace	conditional	0.05
0.277	900	0.5	1	Hotelling Lawley Trace	conditional	0.05
0.157	900	0.5	2	Hotelling Lawley Trace	conditional	0.05
0.510	900	0.5	0.5	Hotelling Lawley Trace	conditional	0.05

Download an .json file of your model design.

- This file may be uploaded in GLIMMPSE V3, so you can reproduce and/or modify the power analysis.

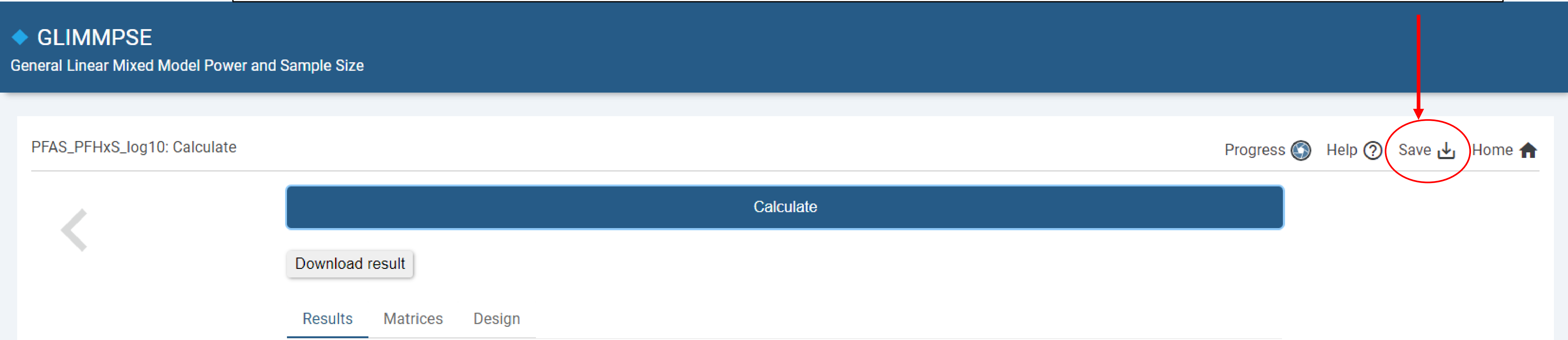

Power	Total Sample Size	Means Scale Factor	Variability Scale Factor	Test	Power Method	Type I Error Rate
0.821	900	1	1	Hotelling Lawley Trace	conditional	0.05
0.510	900	1	2	Hotelling Lawley Trace	conditional	0.05
0.986	900	1	0.5	Hotelling Lawley Trace	conditional	0.05
1.00	900	2	1	Hotelling Lawley Trace	conditional	0.05
0.986	900	2	2	Hotelling Lawley Trace	conditional	0.05
1.00	900	2	0.5	Hotelling Lawley Trace	conditional	0.05
0.277	900	0.5	1	Hotelling Lawley Trace	conditional	0.05
0.157	900	0.5	2	Hotelling Lawley Trace	conditional	0.05
0.510	900	0.5	0.5	Hotelling Lawley Trace	conditional	0.05
